# An immune score reflecting pro- and anti-tumoural balance of tumour microenvironment has major prognostic impact and predicts immunotherapy response in solid cancers

**DOI:** 10.1016/j.ebiom.2023.104452

**Published:** 2023-01-30

**Authors:** Artur Mezheyeuski, Max Backman, Johanna Mattsson, Alfonso Martín-Bernabé, Chatarina Larsson, Ina Hrynchyk, Klara Hammarström, Simon Ström, Joakim Ekström, Siarhei Mauchanski, Salome Khelashvili, Amanda Lindberg, Margrét Agnarsdóttir, Per-Henrik Edqvist, Jutta Huvila, Ulrika Segersten, Per-Uno Malmström, Johan Botling, Björn Nodin, Charlotta Hedner, David Borg, Jenny Brändstedt, Hanna Sartor, Karin Leandersson, Bengt Glimelius, Anna Portyanko, Fredrik Ponten, Karin Jirström, Patrick Micke, Tobias Sjöblom

**Affiliations:** a Department of Immunology, Genetics and Pathology, Uppsala University, Rudbeck Laboratory, 751 85 Uppsala, Sweden; b Department of Oncology-Pathology, Cancer Center Karolinska, Karolinska Institutet, Karolinska vägen, A2:07, 171 64 Solna, Sweden; c City Clinical Pathologoanatomic Bureau, Minsk 220116, Republic of Belarus; d N.N. Alexandrov National Cancer Centre of Belarus, Lesnoy, Minsk, 223040, Republic of Belarus; e Department of Pathology, University of Turku, 20500 Åbo, Finland; f Department of Surgical Sciences, Uppsala University, Akademiska sjukhuset, 751 85 Uppsala, Sweden; g Division of Oncology and Therapeutic Pathology, Department of Clinical Sciences Lund, Lund University, Barngatan 4, 221 85 Lund, Sweden; h Diagnostic Radiology, Department of Translational Medicine, Lund University, Skåne University Hospital, Carl-Bertil Laurells gata 9, 20502 Malmö, Sweden; i Cancer Immunology, Department of Translational Medicine, Lund University, J Waldenströms gata 35, 214 28 Malmö, Sweden

**Keywords:** Tumour immunology, Macrophages, Immunoscore

## Abstract

**Background:**

Cancer immunity is based on the interaction of a multitude of cells in the spatial context of the tumour tissue. Clinically relevant immune signatures are therefore anticipated to fundamentally improve the accuracy in predicting disease progression.

**Methods:**

Through a multiplex *in situ* analysis we evaluated 15 immune cell classes in 1481 tumour samples. Single-cell and bulk RNAseq data sets were used for functional analysis and validation of prognostic and predictive associations.

**Findings:**

By combining the prognostic information of anti-tumoural CD8^+^ lymphocytes and tumour supportive CD68^+^CD163^+^ macrophages in colorectal cancer we generated a signature of immune activation (SIA). The prognostic impact of SIA was independent of conventional parameters and comparable with the state-of-art immune score. The SIA was also associated with patient survival in oesophageal adenocarcinoma, bladder cancer, lung adenocarcinoma and melanoma, but not in endometrial, ovarian and squamous cell lung carcinoma. We identified CD68^+^CD163^+^ macrophages as the major producers of complement C1q, which could serve as a surrogate marker of this macrophage subset. Consequently, the RNA-based version of SIA (ratio of CD8A to C1QA) was predictive for survival in independent RNAseq data sets from these six cancer types. Finally, the CD8A/C1QA mRNA ratio was also predictive for the response to checkpoint inhibitor therapy.

**Interpretation:**

Our findings extend current concepts to procure prognostic information from the tumour immune microenvironment and provide an immune activation signature with high clinical potential in common human cancer types.

**Funding:**

Swedish Cancer Society, Lions Cancer Foundation, Selanders Foundation, P.O. Zetterling Foundation, U-CAN supported by SRA CancerUU, Uppsala University and Region Uppsala.

**Research in context**

**Evidence before this study**

Tumours are composed of malignant cells embedded in a microenvironment of host tissue elements: collagen and elastic fibres, mesenchymal cells, endothelial cells, fibroblasts and infiltrating immune cells. These infiltrating immune cells can support or suppress tumour progression and influence response to anti-cancer treatments.

The initial interest was restricted to cytotoxic T cells, considered as major cancer cell killers, and the immune scoring system, Immunoscore®, which evaluates the abundance of T-lymphocytes in cancers tissue, was established for survival prediction in colon cancer. However, recent research has supported key roles of other immune cell classes in different cancer types, including NK cells, T-helpers, dendritic cells, and macrophages.

Following advances in the understanding of the anti-tumoural function of the immune system, a novel class of immunotherapy drugs—immune checkpoint inhibitors—was discovered and has revolutionized the treatment of several cancer types. However, while some tumours are sensitive to immune checkpoint inhibitors, others are initially refractory or develop resistance. This leads to decreased quality of life due to recurrent disease or drug toxicity, increased patient mortality, and increased economic burden on the health care system. Thus, the establishment of reliable immune biomarkers for prognosis and prediction of response to immunotherapy is an urgent need in modern oncology. However, the progress in the field is slowed down by the inherent complexity of the immune system, which requires advanced methods for the quantitative analysis of the immune cell in clinical samples.

**Added value of this study**

We generated a comprehensive overview of the immune landscape in colon cancer by *in situ* analysis of 15 distinct subclasses of T- and B-lymphocytes, NK cells and macrophages. This analysis identified the immune cell signature with the highest prognostic value, which included scores of T-lymphocytes and a subset of macrophages. We demonstrated unique features of these macrophages, suggesting them as potential therapeutic targets. Further, we validated the prognostic ability of this signature in four other tumour types by *in situ* analysis and confirmed our findings using independent datasets. Finally, we demonstrated its ability of the signature to predict response to immunotherapy.

**Implications of all the available evidence**

We have identified an immune cell marker-defined signature with strong prognostic impact in at least five main solid tumour types and a response predictive relevance in three tested tumour types. We believe that this relatively simple metric of two complementing cell types has potential to become an important parameter for clinical trials and in the diagnostic routine of pathology.

## Introduction

Tumours are composed of malignant cells embedded in a microenvironment of extracellular matrix, resident mesenchymal cells, endothelial cells, and infiltrating immune cells.^1^^,^^2^ These host elements of the tumour microenvironment support or suppress tumour progression and influence response to anti-cancer treatments.^3^, ^4^, ^5^ In colorectal cancer an immune scoring system Immunoscore® was established and surpassed traditional clinical parameters such as T and N stage for prediction of prognosis in stage I-III colon cancer patients. It evaluates the abundance of CD3^+^ and CD8^+^ T cells in immunohistochemically stained slides from patient tissue.^6^, ^7^, ^8^ However, recent findings support roles for other immune cells, including T-regulatory lymphocytes, B cells, NK cells, dendritic cells and macrophages, in cancer progression.^9^, ^10^, ^11^, ^12^, ^13^, ^14^ Tumour associated macrophages (TAMs) are the most abundant immune cells in the microenvironment of many solid tumours, with TAM subsets exerting anti-tumoural as well as tumour promoting activity.^15^, ^16^, ^17^, ^18^, ^19^, ^20^ Thus, clinically relevant immune signatures which consider both pro- and anti-tumoural cell types can be anticipated to fundamentally improve the accuracy in predicting disease progression.

The immune microenvironment has particularly become in focus of cancer research with the introduction of immune checkpoint inhibitors (ICI) that have revolutionized the treatment in several cancer types.^21^ While some patients are sensitive to ICI and show impressive treatment outcomes, others are initially refractory or develop resistance. Current response-predictive biomarkers for ICI mainly include features of the tumour cells, such as PD-L1 expression, mismatch repair deficiency and tumour mutation burden (TMB).^22^, ^23^, ^24^ However, the predictive accuracy of these biomarkers is only modest.^25^ As high costs and significant adverse effects are drawbacks of ICIs, reliable biomarkers for prediction of therapy response is an urgent need in oncology. The immune cell composition as analysed by multiplex immunohistochemistry (mIHC), which combines the advantages of traditional IHC with multi-marker potential, outperformed the hitherto best tumour-cell markers for prediction of response to ICI,^25^ making this a promising method for future clinical use.^26^ Thus, the establishment of novel reliable immune biomarkers for prognosis and prediction of therapy-response to immunotherapy is an urgent clinical need.

Here we generated a comprehensive overview of the immune landscape in colon cancer and dissected the tumour immune microenvironment with a specific focus on pro-tumoural immune cell subclasses and those with potential impact on the resistance to ICI. Specifically, we (1) map the immune landscape in colon cancer by *in situ* analysis of 15 distinct subclasses of T- and B-lymphocytes, NK cells and TAMs, (2) identify the immune cell signature with the highest prognostic value in colon cancer, (3) demonstrate the prognostic ability of this signature in four other tumour types and (4) demonstrate its ability to predict response to immunotherapy.

## Methods

Supplementary Figure S1 outlines the schematic structure of the study, including study phases, analytical methods and cohorts.

### Ethics

Seven tissue microarray (TMA) cohorts were obtained from research centres in Sweden and Finland within ethical permits from the regional ethical committees.

- *colorectal cancer cohort:* approved by the regional ethical committee in Uppsala, Sweden (2010/198 and 2015/419);
- *melanoma* cohort: approved by the regional ethical committee in Uppsala, Sweden (2005/232);
- *lung cancer* cohort: approved by the regional ethical committee in Uppsala, Sweden (2012/532);
- *gastroesophageal cancer* cohort: approved by the regional ethical committee in Lund (2007/445);
- *urothelial cancer* cohort: approved by the regional ethical committee in Uppsala (2005/143);
- *uterine corpus endometrial carcinoma* cohort: approved by the ethical review board in Helsinki (2016/010);
- *ovarian carcinoma* cohort: approved by the regional ethical committee in Lund (2007/445);

For more details see Table 1, Supplementary Tables S1–S3.

**Table 1:** Resource table.

Reagent or resource	Source	Identifier or reference
**Study cohorts**		
Colorectal cancer	Uppsala-Umeå Comprehensive Cancer Consortium	^27^
Melanoma	Uppsala region, Sweden	^28^
Lung Cancer	Uppsala University Hospital, Sweden	^29^
Urothelial cancer	Uppsala University Hospital	^30^
Gastroesophageal cancer	University Hospitals of Lund and Malmö	31, 32, 33
Ovarian carcinoma	The Malmö Diet and Cancer Study and the Malmö Preventive Project	^34^
Uterine corpus endometrial carcinoma	Turku University Hospital, Finland	^35^^,^^36^
**Deposited data**		
Single cell RNA-seq data, colorectal cancer	GEO	GEO: GSE144735
Single cell RNA-seq data, lung cancer	ENA	ENA: ERP110453
Single cell RNA-seq data, uveal melanoma	GEO	GEO: GSE139829
Single cell RNA-seq data, 15 organs	GEO	GEO: GSE159929
Single cell RNA-seq data, ICI treated melanoma	GEO	GEO: GSE120575
Bulk RNA data, ICI treated melanoma	GEO	GEO: GSE78220
Single cell RNA-seq data, ICI treated Renal Cell Carcinoma	Single Cell Portal	dbGaP: phs002065.v1.p1 https://singlecell.broadinstitute.org/single_cell/study/SCP1288/tumor-and-immune-reprogramming-during-immunotherapy-in-advanced-renal-cell-carcinoma#study-summary
**Antibodies**		
Mouse monoclonal anti-CD8a	Thermo Fisher	Clone C8/144B
Mouse monoclonal anti-CD4	Agilent	Clone 4B12
Mouse monoclonal anti-CD20	Agilent	Clone L26
Rabbit polyclonal anti-FoxP3	Cell Signaling	Clone D6O8R
Mouse monoclonal anti-CD45RO	Thermo Fisher	Clone UCHL1
Mouse monoclonal anti-PanCK	Abcam	Clone C-11
Mouse monoclonal anti-Cytokeratin	Agilent	Clone AE1/AE3
Mouse monoclonal anti-E-cadherin	BD Biosciences	Clone 36/E
Mouse monoclonal anti-Melan A	Agilent	Clone A103
Mouse monoclonal anti-CD3	Agilent	Clone F7.2.38
Mouse monoclonal anti-CD56	Agilent	Clone 123C3
Rabbit polyclonal anti-NKp46	Thermo Fisher	Clone NCR1
Mouse monoclonal anti-CD68	Agilent	Clone PG-M1
Mouse monoclonal anti-CD163	Novocastra	Clone 10D6
**Chemicals**		
Spectral DAPI	Akoya Biosciences	Cat# FP1490
Opal 520 Reagent Pack	Akoya Biosciences	Cat# FP1487001KT
Opal 540 Reagent Pack	Akoya Biosciences	Cat# FP1494001KT
Opal 570 Reagent Pack	Akoya Biosciences	Cat# FP1488001KT
Opal 620 Reagent Pack	Akoya Biosciences	Cat# FP1495001KT
Opal 650 Reagent Pack	Akoya Biosciences	Cat# FP1496001KT
1X Plus Automation Amplification Diluent	Akoya Biosciences	Cat# FP1609
AR6 buffer, 10X, 4 x 250 mL	Akoya Biosciences	Cat# AR6001KT
AR9 buffer, 10X, 4 x 250 mL	Akoya Biosciences	Cat# AR9001KT
Antibody Diluent/Block, 1X, 100 mL	Akoya Biosciences	Cat# ARD1001EA
Opal Polymer HRP Ms + Rb, 1X, 50 mL	Akoya Biosciences	Cat# ARH1001EA
ImmPRESS™ HRP Anti-Mouse IgG	Vector Laboratories	Cat# MP-7402-50
ImmPRESS™ HRP Anti-Rabbit IgG	Vector Laboratories	Cat# MP-7401-50
ProLong™ Diamond Antifade Mountant	Thermo Fisher	Cat# P36961
Opal Staining Jar, 4-pack	Akoya Biosciences	Cat# STJAR4
**Software and algorithms**		
RStudio	RStudio Team (2020). RStudio: Integrated Development for R. RStudio, PBC, Boston, MA	https://www.rstudio.com
Seurat (R package)	Hao∗, Hao∗ et al., Cell 2021^37^	https://satijalab.org/seurat/
inForm Tissue Analysis Software	Akoya Biosciences	inForm®
**Other**		
Phenoimager HT system	Akoya Biosciences	Phenoimager™

Information about patient materials, instruments and reagents used in the study.

### Study cohorts and tissue microarrays

*The colorectal cancer (CRC) cohort* consists of prospectively collected CRC patients living in Uppsala County, Sweden, most of whom have been included in the Uppsala-Umeå Comprehensive Cancer Consortium (U-CAN).^27^^,^^38^ In total, 937 patients were diagnosed with CRC between 2010 and 2014 in the region. Of them, 746 (80%) were included in a TMA. For the present study, only patients with TMA material from their primary tumours were selected. After the staining procedures and quality control, 497 patients had data from both immune panels (see below in Multiplex immunofluorescence staining for more details) of whom 286 patients had TNM stage I-III operated colon cancer not receiving any treatment prior to the surgery. The clinicopathological characteristics of the included patients and their tumours are presented in Supplementary Tables S1 and S2. All patients received stage-stratified standard of care according to the Swedish national guidelines from 2008. According to the guidelines, colon tumours were recommended primary surgery and adjuvant chemotherapy if risk factors for recurrence were present. If the colon tumour was considered inextirpable/borderline resectable, preoperative chemotherapy was administered to shrink the tumour before surgery, but these tumours were excluded for all analyses of stage I–III. Rectal cancers received preoperative or neo-adjuvant radiotherapy/chemoradiotherapy stratified according to risk for locoregional or systemic recurrence.

The *melanoma* cohort encompassed TMA cores from 94 patients diagnosed with primary cutaneous malignant melanoma in the Uppsala region, Sweden, from 1980 to 2004^28^ (Supplementary Table S3).

The *lung cancer* cohort encompassed TMA cores from 163 patients diagnosed with adenocarcinoma and 89 patients diagnosed with squamous cell carcinoma, who underwent surgical treatment at Uppsala University Hospital, Sweden from 2006 to 2010^29^ (Supplementary Table S3).

The *gastroesophageal cancer* cohort included TMA cores from 121 patients with chemoradiotherapy-naïve gastroesophageal adenocarcinomas who underwent surgery at the University Hospitals of Lund and Malmö from 2006 to 2010^31^, ^32^, ^33^ (Supplementary Table S3).

The *urothelial cancer* cohort encompassed TMA cores collected from primary urothelial tumours from 224 patients undergoing surgery at Uppsala University Hospital between 1984 and 2005^30^ (Supplementary Table S3).

The *uterine corpus endometrial carcinoma* cohort consisted of TMA cores from 295 uterine carcinomas from patients surgically treated at Turku University Hospital, Finland, between 2004 and 2007^35^^,^^36^ (Supplementary Table S3).

The *ovarian carcinoma* cohort was presented as TMA cores from invasive ovarian cancer cases, derived from two pooled prospective, population-based cohorts; the Malmö Diet and Cancer Study and the Malmö Preventive Project^34^ (Supplementary Table S3).

Patient data, including patient sex was collected from the clinical records. Patient gender was not assessed in this study.

Formalin-fixed paraffin-embedded tissue blocks of primary tumours were used to construct TMAs. In the CRC cohort, each case was represented on the TMA with cores derived from the central part of the tumour and from the invasive margin. In the other cohorts, representative tumour areas without visually identified large necroses or fat tissue regions were selected for TMA construction.

### Multiplex immunofluorescence staining and imaging

For the mIHC, 4 μm thick TMA sections were de-paraffinized, rehydrated and rinsed in distilled H_2_O. Two staining protocols were established for the two panels of antibodies: the lymphocyte panel, with CD4, CD8, CD20, FoxP3, CD45RO, and pan-cytokeratin (CK), and the NK/macrophage panel encompassing CD56, NKp46, CD3, CD68, CD163, and pan-CK, as previously described^39^^,^^40^ (Table 1, Supplementary Table S4). The stained TMAs were imaged using the Phenoimager HT system (Akoya) in multispectral mode at a resolution of 2 pixels/μm.

### Image analysis and thresholding

The multi-layer multispectral image (Supplementary Figure S2a) was processed through a spectral unmixing algorithm to generate an oligo-layer image, where one grey-scale layer corresponded to either specific staining, DAPI or tissue autofluorescence. For visualisation purposes the grey-scale layers were assigned different colours and demonstrated as multi-colour image Supplementary Figure S2a. The vendor-provided machine learning algorithm, implemented in the inForm software, was trained to split tissue into three categories: tumour compartment, stromal compartment, or blank areas (Supplementary Figure S2c). The training was performed for each cohort separately by providing a set of the samples that was manually annotated by pathologists. Blank tissue was removed from the ensuing analysis. Relative areas of tumour compartment and stromal compartment varied between cases in each tumour type and between tumour types, as we described before.^41^ In the current study, we did not consider the tumour compartment and the stromal compartment separately. Cell segmentation was performed using DAPI nuclear staining as described.^39^^,^^40^ The perinuclear region at 3 μm (6 pixels) from the nuclear border was considered the cytoplasm area (Supplementary Figure S2d). The cell phenotyping function of the inForm software was used to manually define a representative subset of cells positive to expression of each of the markers and a subset of cells negative to all markers. The intensity of the marker expression in selected cells was used to set the thresholds for marker positivity. Each of the images was manually reviewed and curated by a pathologist to exclude artefacts, staining defects and accumulation of immune cells in necrotic areas and intraglandular structures. The accuracy of tissue segmentation and cell segmentation was also controlled and TMA cores or regions with inadequate segmentation were removed manually. Importantly, we have removed all the regions of necrotic tissues and so-called ‘debris’: the accumulation of necrotic and apoptotic cells in luminal structures in adenocarcinomas. Debris usually contains also immune cells, most of which are macrophages and most of them are CD68^+^CD163^+^. The glandular (in normal tissue) or pseudo-glandular (in adenocarcinomas) lumen is not a part of the tissue. It is an external environment, similar to, for example, intestine microbiota or air in the alveoli. Therefore, to enable best data quality, these regions must be excluded from the analyses (Supplementary Figure S2e).

Each patient/tumour was represented by one to four TMA cores. The tumours of a certain type had the same core diameter which ranged from 0.8 to 1.5 mm. For patients/tumours which were represented by more than one core, the total cell number and the total tissue area from all available cores were used for the computation of immune cell densities and for the generation of SIA. Final analysed tissue areas are visualised in Supplementary Figure S3a. Mean (median) tissue area across all analysed cohorts was 1.84 (1.88) mm^2^ and ranged from minimal 15,720.39 μm^2^ in melanoma to maximal 6.51 mm^2^ in colon adenocarcinoma. The absolute cell counts for CD8^+^, CD68^+^CD163^−^, CD68^+^CD163^+^, and CD68^−^CD163^+^ cells, ranged from 0 (all cell types in different cancers) to 15,021, 10,166, 7170 and 19,728 cells respectively (Supplementary Figure S3b).

**Fig. 1:**
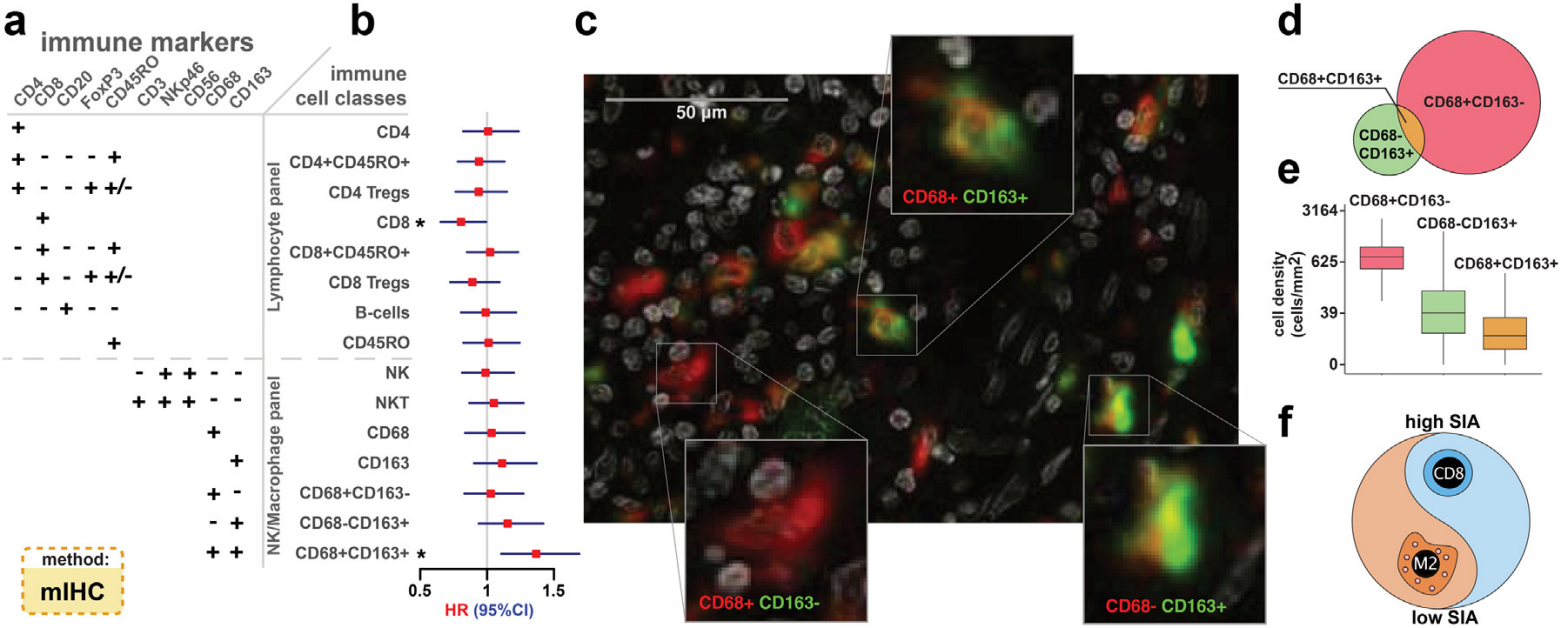
**Prognostic value of CD8**^**+**^**T cells and CD68**^**+**^**CD163**^**+**^**macrophages revealed by comprehensive characterization of immune cell subsets in 286 therapy-naïve colon cancers** (See also Figures S1–S4). **(a)** Immune marker combinations in IHC panels define classes and subclasses of immune cells (See also Supplementary Table S1). **(b)** Forest plot of univariable associations of tissue immune cell densities translated into three-level categorised values, with OS in patients of stage I-III. Filled squares, hazard ratios (HR); whiskers, 95% confidence intervals (CI), ∗p < 0.050 (Cox regression). **(c)** Representative multiplex macrophage marker staining of colon cancer tissue. Expression of two markers, CD68 (red) and CD163 (green) with nuclear DAPI staining (white), visualised in pseudocolours, identified three cell types (insets), M1-like macrophages, M2-like macrophages and CD68^−^CD163^+^ cells. **(d)** Venn diagram of the counts of cells in the entire cohort expressing CD68 only (red, n = 9.0 × 10^5^), CD163 only (green, n = 1.9 × 10^5^) or both markers (gold, n = 4.4 × 10^4^). **(e)** Density of three macrophage subsets in patient tumours. Boxes, median and interquartile range (IQR) of the ratios; whiskers, 1.5 IQR. **(f)** Signature of immune activation (SIA), defined as the ratio of CD8^+^ cell density to the sum of CD8^+^ and M2-like cell densities.

Intensity thresholds for the markers were determined in the R programming environment [R Core Team, 2013] by GeneVia Technologies (Tampere, Finland). The marker-specific thresholds were defined by the distributions of the positive and negative cell intensities for that marker. Marker-specific probability density distributions were estimated by smoothing the intensity values with Gaussian kernel estimation with automatic bandwidth detection using the density function of the R package *stats*. The intensity thresholds for each marker were established as (1) the mean value of the highest intensity of the negative cells and the lowest intensity of the positive cells, if the intensities of the positive and negative cells did not overlap, or (2) as the intensity value which minimised the overall classification error based on the probability density distributions, if there was overlap. The False Positive Rate, True Positive Rate, False Negative Rate, True Negative Rate, and the overall classification error were calculated for each established threshold, i.e. for each marker, and controlled individually. The thresholds were established separately and independently for each tumour type and were applied to the raw output data of the complete cohorts. Every cell was thus characterised as positive or negative for each marker in the panel. This data was used to classify the cell and define its immune subtype (Fig. 1a). Finally, the cell counts were normalised against analysed tissue area size and used as a measure of cell density (units per mm^2^) in downstream analyses: (a) for initial survival analysis the patients were classified as having low, medium or high density of each immune cell subtype, using the 33.3 and 66.6 percentiles as cut-offs (Fig. 1b); (b) the continuous values of CD8 and CD68+CD163+ cell densities were used for the computing of immunosorbent and of the signature of immune activation (see below more details).

To ensure that the TMA cores were representative of the entire tumour, we sectioned tumour blocks (four from colorectal cancer and four from lung cancer) and stained the whole sections with antibodies against CD8, CD68 and CD163. These whole slide sections (WSS) were then imaged using the tiles equivalent to the size of those applied for imaging of the TMA cores (i.e. 3752 × 2808 pix or 1.86 × 1.39 mm), generating from 13 to 93 tiles per WSS (Supplementary Figure S4a). The image analysis pipeline was applied as described above to generate cell density metrics for each tile. We selected one tile at random from each of the WSSs, generating a small in silico TMA cohort, and then compared the distribution of immune cell densities in with corresponding densities in all other tiles generated form the same WSS using the two-sample Kolmogorov–Smirnov test. The two-sample Kolmogorov–Smirnov test examines the maximum difference between the two cumulative distributions, and reports a p-value. Thus, a low p-value indicates significant difference between two analysed datasets while a high p-value suggests that the two datasets are similar. We repeated random selection of tiles for the in silico TMA cohort and Kolmogorov–Smirnov testing 1000 times for each of the markers (Supplementary Figure S4b). At significance level of 0.05, the following fraction of random sampling iterations demonstrated significant difference between the in silico TMA cohort and WSSs: CD8^+^, 0.004; CD68^+^CD163^−^, 0.013; CD68^+^CD163^+^, 0.008; CD68^−^CD163^+^, 0.019. These results strongly support that the TMA images are representative of the tumour tissue from where they were sampled with regards to the markers of relevance for determining the SIA score.

### Signature of immune activation and Immunoscore

The signature of immune activation (SIA) was computed as the ratio of CD8^+^ cell density to the sum of the densities of CD8^+^ and CD68^+^CD163^+^ cells, or SIA = (CD8 density)/(CD8 density + CD68^+^CD163^+^ cell density). For the Immunoscore (IS), each tumour in the CRC TMA cohort was represented by cores derived from the central part and the invasive margin. The CD3 and CD8-positive cells were defined in each of the regions, thus resulting in four values per case (i.e. CD3 density in tumour centre, CD8 density in tumour centre, CD3 density at the invasive margin, CD8 density at the invasive margin). In 13% of cases for CD8 and in 18% cases for CD3 were incomplete, with the cores presented only from one location (i.e., only from tumour centre or invasive margin). The IS was generated as described^6^ by computing a mean of the four values, or as a mean of three values in incomplete cases. In the other cohorts, the TMA cores were obtained from the bulk tumour region, without separation between central parts and invasive margin. Thus, for these tumours two values per case were obtained (CD3 and CD8-positive cell density) and IS was generated by computing a mean of the two. Further, using the mean percentiles, IS was categorised into 3 groups: Low (mean percentile 0–25%), Intermediate (25–70%) and High (70–100%).

### Analysis of bulk RNA data

We used the publicly available bulk RNA dataset from melanoma patients (GEO: GSE78220^42^). The analysis was performed using R. SIA was generated by computing a ratio between the expression of CD8A to either of C1QA, C1QB or C1QC. Only samples taken before ICI therapy were included in the analysis. One patient had two samples, and average SIA value was used as patient reference SIA in this case.

### Analysis of single cell RNA sequencing data

We used five publicly available single-cell RNA-seq datasets from colorectal cancers (E-MTAB-8410^43^), lung cancers (E-MTAB-6653^44^), uveal melanoma,^45^ 15 different non-malignant organs of the same individual (GSE159929^46^), renal cell carcinoma patients treated with ICI (Single Cell Portal: dbGaP: phs002065.v1.p1^47^) and 48 melanoma patients treated with anti-PD1 and/or anti-CTLA4 (GSE120575^48^). The analysis was performed using the R package Seurat v4.0.1. For all datasets the cells that expressed fewer than 250 genes were considered outliers and discarded with the exception for GSE120575 where the threshold of 100 genes was used. Cells that had >0.05% of mitochondrial genes were excluded from analysis. The data from individual patients in each dataset was integrated using the alignment by the ‘anchors’ function in Seurat v4.0.1. The standard Seurat workflow was used to scale the integrated data, find relevant components with principal-component analysis and to visualize the results with UMAP and tSNE. Single-cell differential expression was calculated using Seurat. For each comparison, the list of genes was obtained and differential expression was evaluated using adjusted p value and log2(fold change). The gene expression visualization by heatmaps, violin plots and box plots were performed using the standard Seurat workflow and ggplot2 (v3.3.5) package. To characterize the differentiation status of the CD68^+/−^ CD163^+/−^ cells, we performed the analysis of the expression of the genes, which could be presented as canonical M1 or M2 genes, i.e., M1: CD86, CD40, SOCS1, CXCL9, CXCL10, CXCL11, CXCL12, CCL5, and STAT1; M2: ARG1, MMP1, MMP7, MMP12, CD209, MRC1 (CD206), MARCO. Some of M1- and M2-associated genes were not available for the analysis (iNOS and CD80) or were only detected at low levels (MMP1, MMP7, SOCS1, CXCL9, CXCL10, CXCL11).

The public database and web interface KM plotter http://kmplot.com/analysis/ with mRNA data from bladder, oesophageal, rectal, endometrial cancers,^49^ ovarian cancer,^50^ gastric cancer,^51^ lung adenocarcinoma and lung squamous cell carcinoma^52^ was used to verify the prognostic results by computing a ratio between the mRNA expression level of CD8A and either C1QA, C1QB or C1QC to generate a SIA-like metric and by computing average of expression CD8A and CD3E to generate an IS-like metric. These metrics were then dichotomized by applying optimal cut-off and associated with survival by web interface at KM plotter. The survival analysis was visualised by Kaplan–Meier, hazard ratio (HR), 95% confidence interval (95% CI), and log rank p values were used to evaluate statistical significance.

### Statistics

Statistical analyses were performed using R v3.5.1 and SPSS V20 (SPSS Inc., Chicago, IL). Recurrence-free survival (RFS) was computed as the time from surgery to the first documented disease progression including local recurrence or distant metastases or death due to any reason, whichever occurred first. Overall survival (OS) was the time from surgery to death for any reason. The survival analyses were visualised by Kaplan–Meier. Cox proportional hazards model, and log rank p values were used to evaluate statistical significance.

We computed areas under the receiver operating characteristic curve (AUC) with 1000-fold bootstrap resampling,^53^^,^^54^ in order to assess their distributions in the Cox proportional hazards models. Model performances in these analyses were compared using likelihood ratio tests (R package ‘lmtest’) on the original (not bootstrapped) data.

For the evaluation of the Cox proportional hazards model performance in a time-dependent manner, we have implemented time-dependent area under the curve (tAUC) analysis using the “timeROC” R package.^55^

The relative importance of parameters for the estimation of survival risk was computed by fitting multivariable Cox proportional hazards model with clinical factors, SIA and IS being co-variables for the ‘cph’ function from the ‘rms’ R package and applying the ‘anova’ function to the chp object. The anova function reports a matrix of predictors reflecting the importance of the variables in the model, as measured by Wald chi-square (χ^2^).

The public database and web interface KM plotter http://kmplot.com/analysis/ with mRNA data were used to verify the prognostic results.

### Role of funders

The funders did not have any role in study design, data collection, data analyses, interpretation and manuscript writing.

## Results

### Identification of a prognostic signature of CD8^+^ T cells and CD68^+^/CD163^+^ macrophages in colon cancer

To map the immune cell landscape, we performed multiplex labelling of markers in tumour tissue using two antibody panels each consisting of five immune markers for visualization of adaptive and innate immune cells. The co-expression patterns of these markers allowed for immune cell classification into distinct subgroups (see^12^^,^^39^^,^^40^ and Supplementary Methods) (Fig. 1a, Supplementary Figures S1–S4). Specifically, the monocyte/macrophage lineage was sub-divided into CD68^+^CD163^-^ macrophages, CD68^+^CD163^+^ macrophages and CD68^−^CD163^+^ cells. First, we assessed the cell densities as the number of cells of each subtype per total analysed tissue area and categorised cases using the 33.3 and 66.6 percentiles as cut-offs, thereby classifying cases as low, median or high density for each immune cell subtype. Then, we evaluated the prognostic impact of the densities of the different immune cells in stage I-III colon cancers. Two cell classes demonstrated association with OS, namely CD8^+^ T lymphocytes (positive association, HR = 0.81, 95% CI 0.65–0.99, p = 0.042, *Cox regression*) and CD68^+^CD163^+^ macrophages (negative association, HR = 1.37, 95% CI 1.11–1.69, p = 0.0038, *Cox regression*) (Fig. 1b and c). Across all tumours, the CD68^+^CD163^+^ macrophages constituted only 5% of the CD68^+^ macrophages and 23% of the CD163^+^ cells (Fig. 1d), but demonstrated substantial inter-patient heterogeneity with cell densities ranging from 0 to 1080 cells/mm^2^ of tumour tissue (Fig. 1e). Hypothesizing that the relative infiltration levels of CD8^+^ T lymphocytes and CD68^+^CD163^+^ macrophages capture the interplay between anti- and pro-tumoural aspects of the immune microenvironment, we generated a combined immune biomarker by computing the ratio of CD8^+^ cell density to the sum of the densities of CD8^+^ and CD68^+^CD163^+^ cells, and termed it the ‘Signature of Immune Activation’ (SIA) (Fig. 1f).

### The SIA is an independent prognostic biomarker in colorectal cancer and at least four additional tumour types

**Fig. 2:**
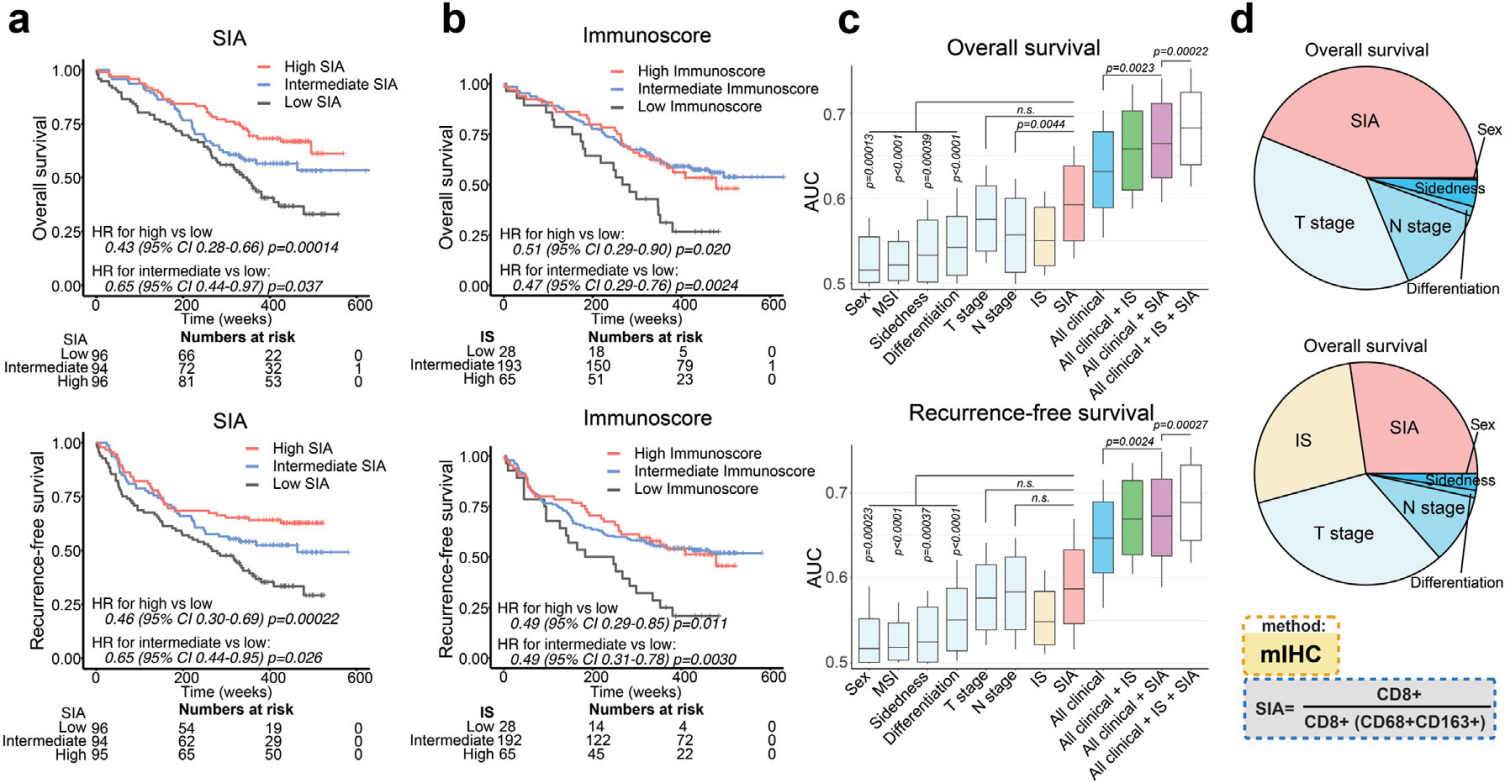
**The SIA is an independent prognostic predictor with performance superior to established clinical and immunological predictors for overall (OS) and recurrence-free (RFS) survival in therapy-naïve colon cancer stage I-III patients** (See also Supplementary Fig. S5 and Supplementary Table S2). **(a)** OS (upper panel) and RFS (lower panel) for the patients (n = 286), stratified into SIA-low, -intermediate and -high groups, with SIA-low used as reference group. **(b)** OS (upper panel) and RFS (lower panel) for the patients stratified by trichotomized IS. Relative hazards were estimated by Cox proportional hazards model in (a) and (b). **(c)** Predictive accuracy of SIA, IS and clinical parameters for OS (upper panel) and RFS (lower panel) using AUC analysis with 1000-fold bootstrap resampling, and the distribution of achieved median values shown in a box plot: horizontal lines indicate 50 percentage, boxes show 95% confidence interval (between 2.5 and 97.5 percentages) and whiskers show upper and lower AUC values. Univariable Cox proportional hazards models were applied to each of the analysed factors separately and multivariable models used to evaluate the impact of factor combinations. The performance of Cox proportional hazards models was compared using the likelihood ratio p value. **(d)** Relative contribution to the prediction of OS of SIA and clinical parameters (upper) or SIA, IS and clinical parameters (lower) determined using the χ^2^ proportion test. SIA, signature of immune activation; IS, immunoscore.

To determine the prognostic value of SIA for OS and RFS, we transformed it into a three-level categorized variable, using the 33.3 and 66.6 percentiles as cut-offs. For comparison with the state-of-art immune scoring system, we generated an Immunoscore-like metric (IS) by quantifying densities of CD3^+^ and CD8^+^ cells at the tumour centre and invasive margin.^6^ Both IS and SIA demonstrated strong associations with OS and RFS in colon cancer stage I–III (Fig. 2a and b). Interestingly, in a multivariable Cox model adjusted for pT stage, pN stage, patient age, sex and MSI status, both SIA and IS were independent predictors for OS and RFS (Table 2). Next, we compared the survival-predictive ability of SIA to IS and established clinical risk factors. AUC^53^^,^^54^ analysis identified T stage as the strongest current predictor for OS (median AUC 0.58) and N stage for RFS (median AUC 0.58) (Fig. 2c). However, median AUC for SIA (0.59 for OS and RFS), was higher than for T stage and N stage although only for N stage in OS the difference reached statistical significance. Combining SIA with clinical parameters improved the survival-predictive ability (median AUC 0.66 and 0.67 for OS and RFS, respectively). Finally, integration of clinical parameters, IS and SIA in the same model resulted in median AUC 0.68 and 0.69 for OS and RFS, respectively (Fig. 2c). The predictive accuracy of IS and SIA was also evaluated by determining the time-dependent AUC (tAUC).^55^ Interestingly, both SIA and IS had the strongest prognostic impact at 300 weeks, with AUC reaching 0.79 for OS and 0.78 and 0.77 for RFS, however no statistically significant difference between the two immune metrics was observed (Supplementary Figure S5a). The relative contribution to OS prediction was higher for SIA than for T and N stage, and when including IS in the model, the relative contribution of SIA and IS exceeded 50%, the immune cell markers thus surpassing the clinical factors (Fig. 2d). The survival analysis was also performed separately for females and males, and demonstrated overall higher statistical significance of SIA in female patients (Supplementary Figure S5b). Additionally, SIA stratified high and low-risk disease in stage II colon cancer patients (*n* = 117) (Supplementary Figure S5c) and in metastatic colorectal cancer (mCRC) patients (*n* = 66) (Supplementary Figure S5d). Thus, SIA demonstrated independent prognostic performance superior to the best clinical predictors (T and N stage), enhanced the multivariable prediction model in patients of stages I-III, and demonstrated prognostic ability in stage II colon cancer and in mCRC patients.

**Table 2:** The SIA score predicts OS and RFS in 286 stage I-III colon cancer patients.

Co-variable	Overall survival		Recurrence-free survival	
	HR (95% CI)	p value	HR (95% CI)	p value^a^
**Unadjusted Cox model, SIA**				
SIA, three-category				
Intermediate vs low	0.65 (0.44–0.97)	0.037	0.65 (0.44–0.95)	0.026
High vs low	0.43 (0.28–0.65)	0.00014	0.46 (0.30–0.69)	0.00022
**Unadjusted Cox model, IS**				
IS, three-category				
Intermediate vs low	0.47 (0.29–0.76)	0.0020	0.49 (0.31–0.78)	0.0030
High vs low	0.51 (0.29–0.90)	0.020	0.49 (0.29–0.85)	0.011
**Multivariable Cox model**				
SIA, three-category				
Intermediate vs low	0.65 (0.42–0.99)	0.047	0.65 (0.43–0.98)	0.037
High vs low	0.52 (0.33–0.81)	0.0040	0.55 (0.36–0.84)	0.0059
IS, three-category				
Intermediate vs low	0.46 (0.27–0.77)	0.0030	0.49 (0.30–0.80)	0.0041
High vs low	0.62 (0.33–1.16)	0.13	0.62 (0.34–1.12)	0.11
T stage				
T2 vs T1	1.31 (0.44–3.95)	0.63	1.12 (0.37–3.36)	0.84
T3 vs T1	1.20 (0.56–2.59)	0.64	1.25 (0.58–2.67)	0.57
T4 vs T1	3.51 (1.52–8.09)	0.0032	2.97 (1.30–6.79)	0.010
N stage				
N+ vs N0	1.74 (1.18–2.57)	0.0048	2.13 (1.47–3.08)	<0.0001
Age				
Age > 75 vs Age ≤ 75 years	3.49 (2.40–5.09)	<0.0001	2.60 (1.82–3.72)	<0.0001
Sex				
Male vs female	0.85 (0.58–1.23)	0.38	0.90 (0.63–1.28)	0.56
MSI status				
MMR deficient vs proficient	0.82 (0.50–1.35)	0.44	0.95 (0.60–1.51)	0.84
MMR missing vs proficient	1.48 (0.59–3.73)	0.41	1.18 (0.47–2.93)	0.73

Relative hazards, estimated in univariable (for SIA and IS separately) and multivariable (SIA, IS and clinical risk factors) Cox proportional hazards models, using OS and RFS as the endpoints.

MSI, microsatellite instability; MMR, mismatch repair; SIA, signature of immune activation; IS, Immunoscore; HR, hazard ratio; CI, confidence interval.

a Wald p value.

**Fig. 3:**
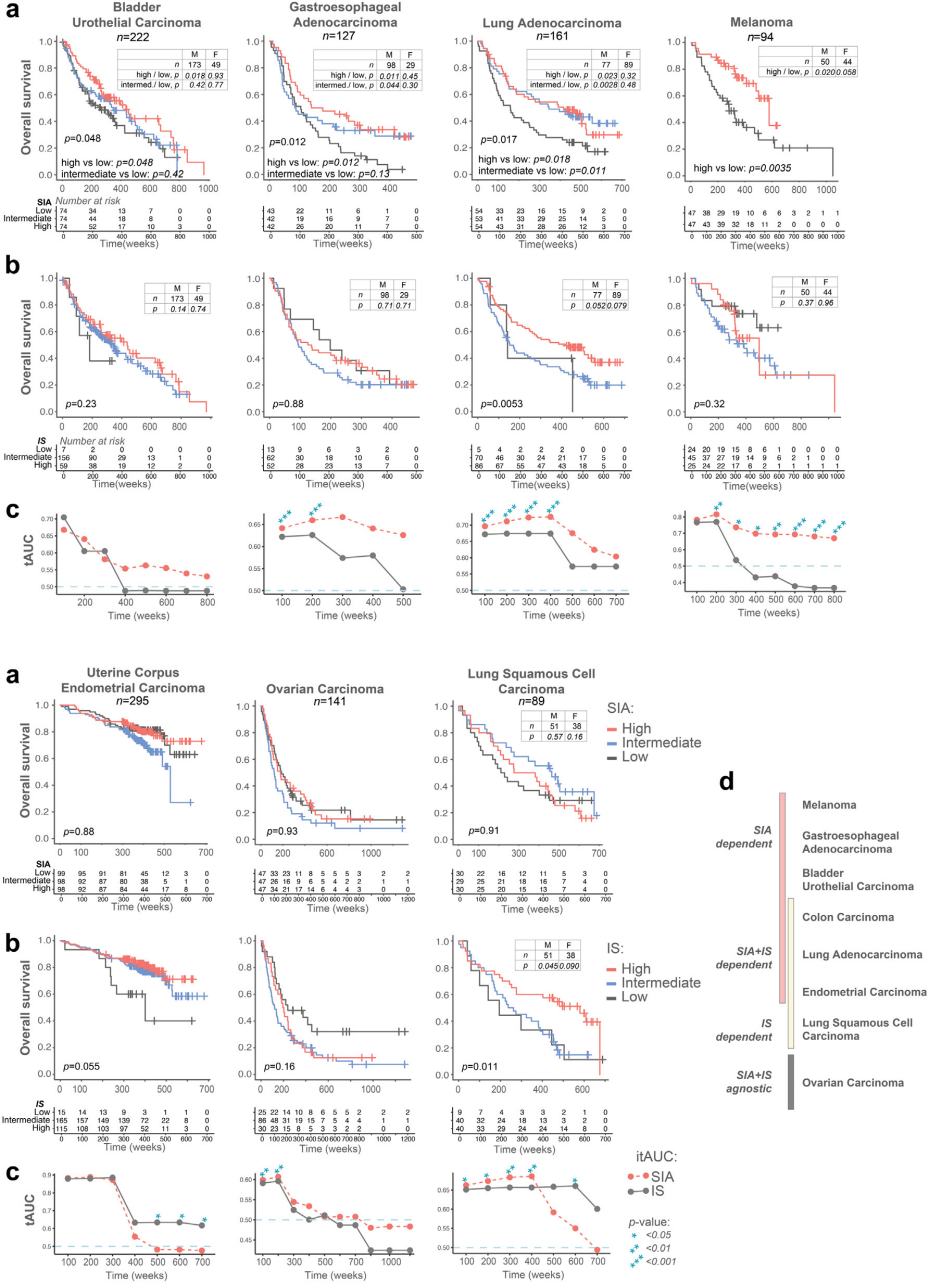
**The SIA is a prognostic predictor in bladder cancer, gastroesophageal cancer, lung adenocarcinoma and melanoma** (See also Supplementary Table S3)**. (a)** Overall survival stratified by SIA in seven tumour types. Tissue microarrays encompassing 94–295 cases of the respective tumour type were stained and the patients in each cohort stratified in terciles according to SIA score, except melanoma, which was stratified in two groups split by the median. Statistical analysis by log-rank test for three groups, and/or Cox regression for pairwise comparison (high vs low and intermediate vs low). Insert tables demonstrate significance of SIA in female or male patients. **(b)** Overall survival stratified by IS in 7 tumour types. Statistical analysis performed by log-rank test for three groups. **(c)** Comparison of the predictive accuracy of IS and SIA for OS in 7 tumour types, generated using tAUC analysis. Statistical analysis performed for the evaluation of the difference between the survival models of AUC and IS: the statistically significant time-points are indicated by asterisks; **(d)** The OS predictive ability of SIA and IS in 8 analysed tumour types.

Next, we asked whether the SIA score was prognostic also in other cancers. We analysed a total of 1129 patients having either melanoma,^28^ lung adenocarcinoma/squamous cell carcinoma,^29^ bladder urothelial cancer,^30^^,^^56^ gastroesophageal adenocarcinoma,^31^, ^32^, ^33^ endometrial^35^^,^^36^ or ovarian cancer.^34^ Patients were stratified in terciles according to SIA, except for melanoma where the median was used since 41% of patients had the highest possible SIA value. High SIA was associated with longer OS in melanoma, lung adenocarcinoma, bladder urothelial cancer and gastroesophageal adenocarcinomas (p = 0.0035–0.048, *log-rank test and Cox regression*) but not in endometrial (p = 0.89, *log-rank test*), ovarian (p = 0.93, *log-rank test*) and squamous cell carcinoma of the lungs (p = 0.91, *log-rank test*) (Fig. 3a). The survival analysis in females and males, where applicable, demonstrated higher statistical significance of SIA in male patients. The IS, when analysed in the same tumours, was prognostic in lung cancers only (both adenocarcinoma and squamous cell carcinoma), and trend was seen in endometrial carcinoma (Fig. 3b). Further, SIA was better than IS for prediction of OS in these cancers, as demonstrated by tAUC analysis (Fig. 3c). Interestingly, the tumour types demonstrated different dependence on the immune signatures, which allowed us to identify four distinct cancer types (Fig. 3d): (a) SIA-dependent/IS-agnostic tumours (melanoma, gastroesophageal adenocarcinomas, bladder urothelial cancer); (b) SIA and IS-dependent tumours (colon cancer, lung adenocarcinoma, endometrial carcinoma); (c) SIA-agnostic/IS-dependent tumours (squamous cell lung carcinoma); (d) SIA and IS-agnostic tumours (ovarian carcinoma). Thus, tumours demonstrated different dependence from the SIA and IS, with SIA being prognostic in at least five tumour types.

### The prognostic CD68^+^CD163^+^ macrophages produce complement complex subunit C1Q

To further define the CD68^+^CD163^+^ cells, we analysed single-cell RNA sequencing data from CRC (n = 54259 cells),^43^ lung cancers (n = 32439 cells)^44^ and uveal melanoma (n = 97550 cells).^45^ We identified three cell subsets, based on gene expression levels of CD68 and CD163 (i.e., CD68^+^CD163^+^, CD68^+^CD163^−^ and CD68^−^CD163^+^ cells) and investigated genes associated with M1 or M2 macrophage differentiation (Supplementary Figure S6). The CD68^+^CD163^+^ cells were characterized primarily by the expression of M2 markers but also some M1 markers, in line with previous findings.^57^ Next, we examined monocyte-related gene expression^58^, ^59^, ^60^, ^61^, ^62^, ^63^ in the three cell subsets. Interestingly, CD68^+^CD163^+^ cells demonstrated high expression of CD14, CD16, ITGAX (CD11c), CD86 and CD45 in all three tumour types, supporting an origin from blood intermediate monocytes^58^ (Supplementary Figure S6, lower panel). The CD68^−^CD163^+^ cells had high CD14 and low or no CD16 expression, suggesting an origin from classical monocytes. Finally, the CD68^+^CD163^−^ cells had low or no expression of CD14, CD16 and CD45, thus lacking monocyte characteristics.

**Fig. 4:**
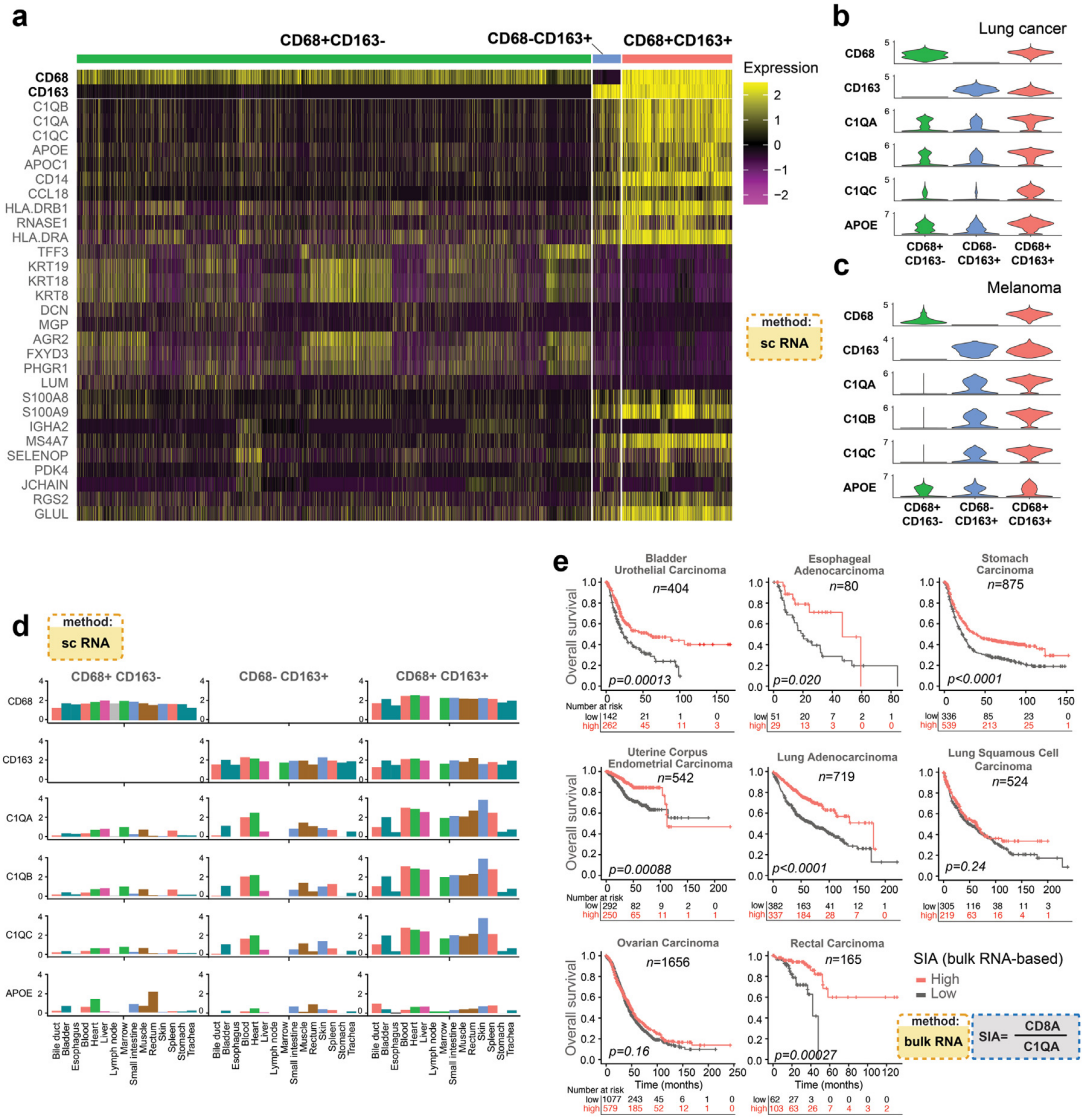
**Complement complex C1q expression is a hallmark of CD68**^**+**^**CD168**^**+**^**macrophages and can be used to generate the SIA signature from bulk RNA sequencing of tumours. (a)** Heatmap of scaled gene expression values for the 29 genes with highest fold difference between CD68^+^CD163^−^, CD68^+^CD163^+^ and CD68^−^CD163^+^ macrophage classes in colorectal cancers^43^ (See also Supplementary Figs. S6 and S7, Supplementary Tables S5–S7). **(b** and **c)** Expression level distributions of the macrophage associated genes C1QA-C and APOE in lung cancer^45^ (B) and uveal melanoma^44^ (See also Supplementary Figs. S8 and S9). **(d)** Gene expression level distributions of C1QA-C and APOE in three subsets of macrophages in 15 non-diseased organs (See also Supplementary Table S8). **(e)** Overall survival stratified by dichotomized ratio between the bulk RNA expression levels of CD8A and C1QA in seven tumour types using gene expression data from the KM plotter database (See also Supplementary Fig. S10).

The analyses of differentially expressed genes in these three subsets of macrophages demonstrated that cells of the CD68^+^CD163^+^ subgroup overexpressed C1QA, C1QB, and C1QC, together encoding C1q, a subcomponent of the C1 complement complex (Fig. 4a, b and c, Supplementary Figure S7, Supplementary Tables S5–S7). In CRC and lung cancer we also observed high expression of APOE, encoding Apolipoprotein E, restricted to CD68^+^CD163^+^ macrophages. By analysis of the complete datasets of three single-cell collections, we observed that C1QA, C1QB, and C1QC (but not APOE) were expressed almost exclusively in macrophages (Supplementary Figure S7), as described.^64^, ^65^, ^66^ Macrophages from tumour and peritumoural tissues in CRC and lung cancer demonstrated comparable levels of expression of C1QA-C and APOE (Supplementary Figures S8 and S9). Then, we explored single cell RNA sequencing data from 15 different non-malignant organs of the same individual^46^ (Supplementary Table S6). Only a small fraction of cells expressed C1QA-C (average 4%), whereas a higher fraction expressed APOE (average 17%). The majority of C1QA-C expressing cells were macrophages (defined by CD68 and/or CD163 positivity) ranging from 45 to 56% of the positive cells in lymph node to 91–93% in liver. When analysing macrophage subclasses, C1QA-C expression was characteristic for CD68^+^CD163^+^ macrophages but very low in CD68^+^CD163^−^ cells (Fig. 4d).

Taken together, CD68^+^CD163^−^, CD68^+^CD163^+^ and CD68^−^CD163^+^ cells demonstrate different gene expression patterns, supporting the tissue-resident nature of the former, and blood monocyte-derived origin for the latter two. The expression of C1q components was particularly characteristic for CD68^+^CD163^+^ macrophages in malignant and normal tissues.

### The ratio of CD8 to C1Q gene expression is prognostic in several tumour types

As the C1QA-C expression in cancers was mainly detected in CD68^+^CD163^+^ macrophages, the synthesis of complement C1q components analysed at the bulk RNA level can potentially be used to estimate the amount of pro-tumoural CD68^+^CD163^+^-like macrophages. We extracted bulk mRNA expression data from the KM plotter database, dichotomized the ratio between the expression level of CD8A and either C1QA, C1QB or C1QC, and performed survival analysis for bladder, oesophageal, rectal, endometrial cancers,^48^ ovarian cancer,^49^ gastric cancer,^50^ lung adenocarcinoma and lung squamous cell carcinoma.^51^ A high ratio was associated with improved survival in all analysed tumour types except lung squamous cell carcinoma and ovarian cancer (Figs. 4e and S10), largely confirming the results from the mIHC based SIA score (Fig. 3 and Supplementary Figure S5). Although it is not possible to reconstruct the original IS metric from bulk RNA data, we generated an IS-like metric, by computing the average of CD8A and CD3E expression and dichotomising the cases into IS-like high and IS-like low. Importantly, an IS-like metric generated from bulk RNA datasets had inferior performance, except for endometrial carcinoma (Supplementary Figure S10). The analysis largely confirmed the initial observation concerning the performance of the SIA and IS in different tumour types (compare Fig. 3d and Supplementary Figure S10). Taken together, the ratio between CD8A and either C1QA, C1QB or C1QC in bulk tumour gene expression data is prognostic in at least five tumour types.

### SIA predicts response to immune therapy

**Fig. 5:**
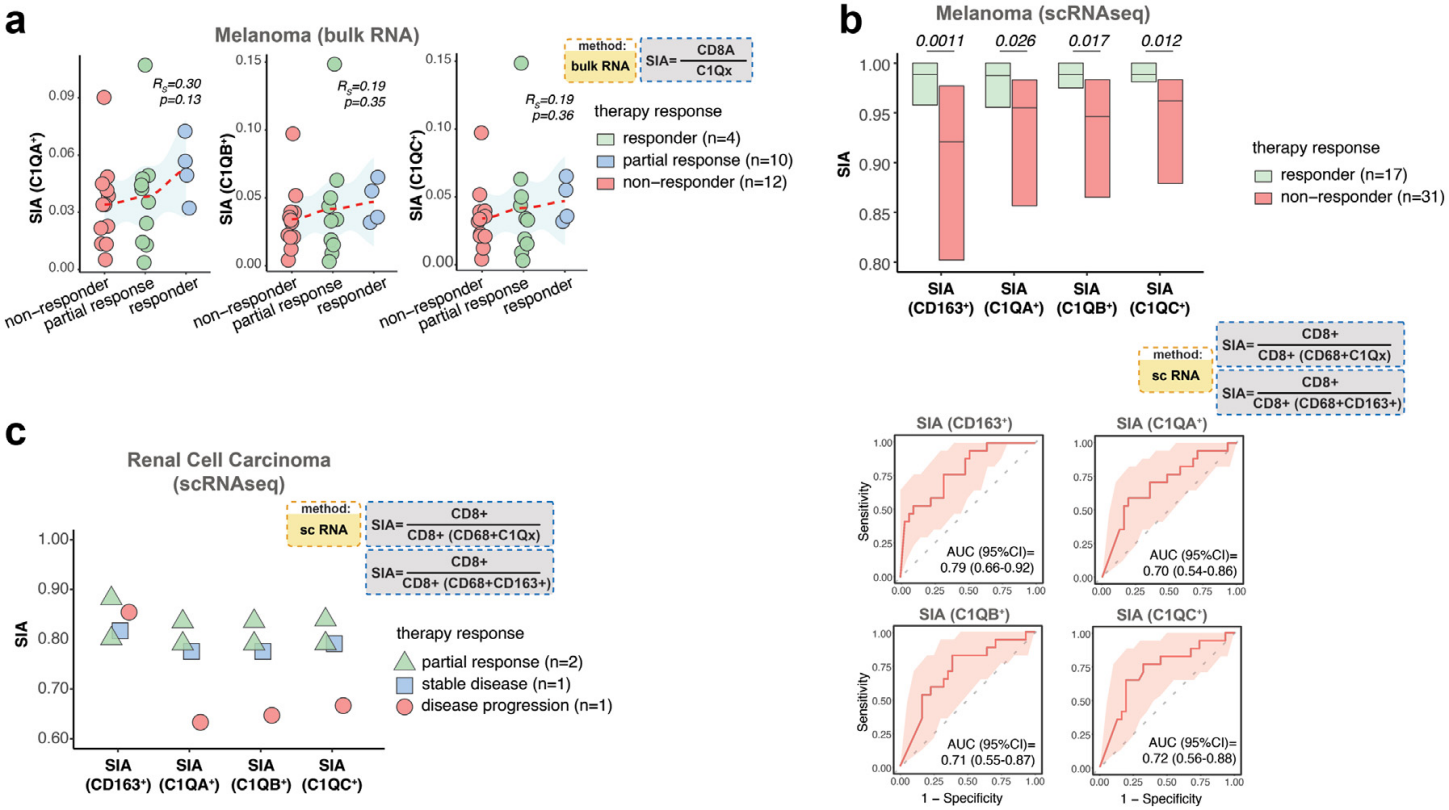
**The SIA predicts response to immune therapy. (a)** SIA values generated from bulk RNA data^42^ by computing the ratio between counts of CD8A and C1QA-C expression in 26 ICI-treated melanomas from patients grouped by response. Spearman-rank correlation was used to test the associations between SIA levels and response. The regression line is visualised by a red dashed line. **(b)** Upper panel demonstrates the difference in SIA values, computed from single cell RNA sequencing data^48^ and based on the ratio between counts of CD8^+^ cells macrophages in responder and non-responder lesions of 48 melanoma patients who received ICI treatment. M2 like macrophages were defined by double-positivity of CD68 and either CD163 or C1QA-C. Lower panel demonstrates the receiver operating characteristics (red line) and 95% confidence intervals (pink areas), calculated for SIA and therapy response **(c)** The difference in SIA values, computed from single cell RNA sequencing data^47^ as the ratio between counts of CD8^+^ cells macrophages in four renal cell carcinoma patients, with different therapy. Horizontal lines indicate median values and boxes show interquartile range. Mann–Whitney U-test was used for statistical analysis.

Finally, we sought to investigate if SIA can discriminate responders for ICI therapy. We analysed bulk RNA from melanomas in patients treated with anti-PD-1 therapy.^42^ We then computed the ratio between CD8A and either C1QA, C1QB or C1QC gene expression. Interestingly, the complete responders (n = 4) had higher SIA values compared to partial responders (n = 10) and non-responders (n = 12) (Fig. 5a). To enable more accurate signature estimation, we next analysed single-cell sequencing data from melanoma patients treated with anti-PD1 and/or anti-CTLA4 (n = 48)^48^ and computed SIA using single cell gene expression levels of CD8A to define CD8^+^ cells and a combination of the expression of CD68 and either CD163, C1QA, C1QB or C1QC to define M2-like macrophages. A clear association between high SIA scores and response to immune check-point inhibitor therapy was observed with SIA derived from CD8A and CD68^+^CD163^+^ (p = 0.0011), CD68^+^C1QA^+^ (p = 0.026), CD68^+^C1QB^+^ (p = 0.017) or CD68^+^C1QC^+^ (p = 0.012, Mann–Whitney U-test), respectively (Fig. 5b). To verify the accuracy of SIA for the prediction of treatment response we performed ROC analysis, which yielded AUCs ranging from 0.70 (for CD8A and CD68^+^C1QA^+^) to 0.79 (for CD8A and CD68^+^CD163^+^). Next, we analysed single-cell RNA sequencing data from renal cell carcinomas,^47^ of which four patients received ICI therapy and had an objective response. Two patients with partial response had higher SIA, derived from cell counts considering complement co-expression by CD68^+^CD163^+^ macrophages, in comparison to one patient with tumour progression (Fig. 5c). Together, these observations indicates that SIA can predict response to ICI treatment in melanomas and potentially other tumour types.

## Discussion

The relationships between cancer cells and host elements are pivotal in tumour development and progression, and immune cells are considered among the most important actors in the tumour microenvironment. In this study we dissected the immune landscape of colon cancer, identified the most prominent pro- and anti-tumoural immune cell subclasses and constructed a biomarker signature with prognostic and response-predictive capacity. Multiplex *in situ* tissue analysis was essential, not only confirming the prognostic impact of CD8^+^ cell infiltration, but also identifying a prognostic subset of CD68^+^CD163^+^ macrophages that was undetectable using a single-marker IHC approach. The relative level of anti-tumoural CD8^+^ cells to the specific subset of CD68^+^CD163^+^ macrophages provided a metric reflecting both pro- and anti-tumoural immunity, which we termed SIA. SIA was independent from other established prognostic parameters and strongly prognostic in several of the most common cancer types. Notably, the prognostic impact of SIA was superior to that of Immunoscore. Finally, the SIA score was also predictive for immunotherapy response. Taken together, we strongly believe that the combination of the two immune cell metrics reflects different aspects of tumour immunity and therefore will increase the accuracy of survival and therapy response prediction.

In many cancer types, specific immune cell infiltration is associated with patient outcome. This includes colon cancer, melanoma, ovarian cancer, lung cancer and subtypes of breast cancer.^66^ The initial interest in immune phenotypes was mainly focusing on cytotoxic T cells, because they were considered as major cancer cell killers, but recent research has indicated key roles of many other immune cells, including natural killer cells, different subsets of T-helpers, dendritic cells and macrophages.^9^, ^10^, ^11^, ^12^, ^13^, ^14^ Distinct subsets of these diverse cell lineages support tumour progression, suppress anti-tumoural immunity and may modulate response to different therapy modalities.

Immunoscore®, which evaluates the abundance of the CD3^+^ and CD8^+^ cells, was validated as an independent prognostic factor in colon cancer stage I–III^6^^,^^7^ and surpassed established clinical parameters such as T and N stage. However, despite the proven validity of the Immunoscore® in colon cancer, the impact of its components has been questioned^67^ considering the broad spectrum of cells identified by CD3-positivity. Thus, the T helper type 2 (Th2) subsets can dominate in tumours and support tumour growth by promoting angiogenesis and inhibiting anti-tumour immunity.^68^ Additionally, Th2 cells drive polarisation of macrophages towards the M2 type and eventually create an immune-suppressive tumour microenvironment.^69^ This implies that the absolute count of CD3^+^ lymphocytes is not sufficient. On the contrary, macrophage polarisation can be captured by well-established methods and, when combined with CD8^+^ lymphocytes, can provide a robust metric to characterise the status of the immune microenvironment. Further, evidence for the prognostic significance of Immunoscore® in other tumour types than colon cancer is limited suggesting that the impact of immune microenvironment on tumour progression, may differ in different cancers. Our findings support this hypothesis and demonstrate different impact on survival of SIA and Immunoscore in different cancer types.

Single-cell-resolution studies have demonstrated multiple subtypes of TAMs and confirmed that M1 and M2 are not two distinct macrophage states, at least not in cancers.^43^^,^^70^ In agreement with these findings, the TAM component in SIA, the CD68^+^CD163^+^ cells in our study were characterised primarily by the expression of M2 markers but also expressed some M1 markers, illustrating the shortcomings of the M1/M2 classification in the cancer context. We performed a series of in-depth analyses of single cell RNA sequence data to characterise the origin of the cells identified by CD68 and/or CD163 positivity in cancer tissue. First, the expression of CD45 (PTPRC), which is a marker for all hematopoietic cells except mature erythrocytes and platelets was observed in all three cell subsets in Lung scRNA data, while in CRC and in Melanoma scRNA datasets it was mostly seen in CD68^+^CD163^+^ cells, lower in CD68^−^CD163^+^ and absent in CD68^+^CD163^−^ (Data not shown). To the best of our knowledge, that could indicate either loss of CD45 during the differentiation of CD68^+^CD163^−^ cells in tumour tissue or their origin from tissue-resident macrophages, while CD68^+^CD163^+^cells, at least partially, demonstrate they origin from blood monocytes. Next, the CD68^+^CD163^+^ (M2-like, component of SIA) cells had high expression of CD14, CD16, ITGAX (CD11c) and CD86 in all three scRNA datasets (Lung, Melanoma, CRC), thus supporting an origin from ‘intermediate’ monocytes,^57^, ^58^, ^59^, ^60^, ^61^, ^62^ although we cannot exclude the increase of the expression of these proteins as a response to local (tissue) stimuli. The CD68^−^CD163^+^ cells had CD14 in all datasets and no (in CRC and Melanoma) or low (Lung) CD16 expression, which indicates an origin from ‘classical’ monocytes.^58^, ^59^, ^60^, ^61^, ^62^, ^63^ Finally, CD68^+^CD163^−^ (M1-like) cells had low (Lung) or no (CRC and Melanoma) expression of both CD14 and CD16, thus lacking monocyte characteristics and suggesting their origin from tissue-resident macrophages or higher differentiation stage of blood-derived monocytes. Interestingly, we did not find evidence that ‘non-classical’ blood monocytes developed into tissue TAMs.

The analysis of CD68^+^CD163^+^ cells in SIA demonstrated that these cells were the main source of complement C1q. Complement C1q plays a prominent role in the clearance of apoptotic cells^71^ and is synthesized predominantly by tissue macrophages and dendritic cells in non-cancer conditions.^64^, ^65^, ^66^ The induction of C1q synthesis in response to injury leads to macrophage differentiation towards an M2-like phenotype.^72^ Additionally, C1q may have a direct suppressive function on cytotoxic CD8^+^ T cells by modulating their mitochondrial metabolism.^73^ Recently, several studies have suggested a tumour-promoting role of C1q independent of classical pathway activation.^65^^,^^74^ With this background it is likely that C1q production in TAM has an immunosuppressive effect and, when integrated into our immune signature, is associated with shorter patient survival in several cancer types.

Although the introduction of ICI that have revolutionized the treatment in several cancer types,^21^ only approximately 20–30% of ICI-treated patients demonstrate disease regression.^75^ Moreover, the treatment is associated with severe side effects and financial burden.^76^^,^^77^ Although the use of PD-L1 expression, mismatch repair deficiency, tumour mutation burden and gene expression profiling for the prediction of response lead to increased number of responders, the predictive accuracy is still only modest. Thus, the AUC for PD-L1 expression, gene expression profiling and tumour mutation burden reached 0.65, 0.65 and 0.69 respectively, as was summarised by Lu et al.^25^ The authors showed that mIHC methods can provide better prediction accuracy (AUC = 0.79), which is comparable to the SIA, demonstrated in our study.

Different strategies targeting the immune modulating capacity of macrophages have been tested in clinical trials with mixed results.^78^, ^79^, ^80^, ^81^, ^82^, ^83^, ^84^, ^85^, ^86^, ^87^, ^88^ This may reflect the divergence of immune activation pathways in tumours, inefficient targeting of key macrophage subpopulations and clearly highlight the need for predictive biomarkers. Our results suggest that SIA should be evaluated in prospective studies to determine its ability in selecting patients that are likely responders to different ICI therapies, and define patient subgroups for combination therapy with anti-macrophage treatments supplementing ICI.

In summary, we have identified an immune cell signature combining CD8 cytotoxic T-cells and a distinct TAM subset reflecting anti- and pro-tumourigenic immune reactions. This marker-defined signature had a strong prognostic impact in at least five main solid tumour types and a response predictive relevance in three tested tumour types. We believe that this relatively simple metric of two complementing cell types has the potential to become an important parameter for clinical trials and in the diagnostic routine of pathology.

### Caveats and limitations

This study was performed on retrospectively collected tissue material and on retrospective RNA sequencing datasets which could potentially introduce bias. For example, the comparison between SIA and IS was initially performed in the same cohort which was used for SIA discovery, and which thus has bias towards SIA impact. However, the replication in bulk RNA data from independent cohorts would argue against this being the case.

Second, the treatment-predictive performance of the SIA was evaluated on datasets with limited size and further validation in larger cohorts is fundamental.

Last, the exact mechanism of the pro-tumoural function of CD68^+^CD163^+^ macrophages is not yet understood.

## Contributors

Conceived study: A.M., P.M., T.S.; Designed experiments: A.M., K.L. B.G., P.M., T.S.; Performed experiments: A.M., M.B., A.L., S.S.; Image curation: I.H., S.M., S.K.; TMA-cohort construction: P.-U.M. (bladder); J.Bo., P.M. (lung); B.N., F.P. (CRC, melanoma); P.M. (lung); K.J. (ovarian, gastroesophageal); Patient database curation: J.M., K.H., M.A. (melanoma); P.-H.E., J.H. (endometrial); U.S. (bladder); P.-U.M. (bladder); J.Bo., P.M., J.M. (lung); K.J., C.H., D.B. (gastroesophageal); K.J., H.S., J.Br., B.N. (ovarian); Data analysis: A.M., A. M.-B., J.E.; Data interpretation: A.M., P.M., T.S.; Wrote the manuscript: A.M., C.L, K.L., B.G., A.P., P.M., T.S. All authors read, revised and approved the final manuscript. A.M. and T.S. have verified the underlying data.

## Data sharing statement

All data reported in this paper will be shared by the lead contact upon request.

All original code has been deposited at [https://github.com/ArturMezh/SIA.git] and is publicly available as of the date of publication. DOIs are listed in Table 1.

Any additional information required to reanalyse the data reported in this paper is available from the lead author upon request.

## Declaration of interests

A.M. and T.S. are co-inventors on a provisional patent application P42105124SE00 “Novel biomarker” regarding a method for the prognosis of survival time of a subject diagnosed with a cancer described herein. K.L. is a board member of Cantargia AB, a company developing IL1RAP inhibitors. This does not alter the Author's adherence to all guidelines for publication.

No other funding except listed in the section Methods/Funders was involved. No other conflicts of interest were disclosed by the other authors.
