# Supplementary material for: An immune score reflecting pro- and anti-tumoural balance of tumour microenvironment has major prognostic impact and predicts immunotherapy response in solid cancers
Source: eBioMedicine. 2023 Jan 30;88:104452. doi: 10.1016/j.ebiom.2023.104452 (PMC9918750; doi:10.1016/j.ebiom.2023.104452)
Supplement: Supplemental Figures and Tables [file mmc1.pdf]

Supplementary Figures

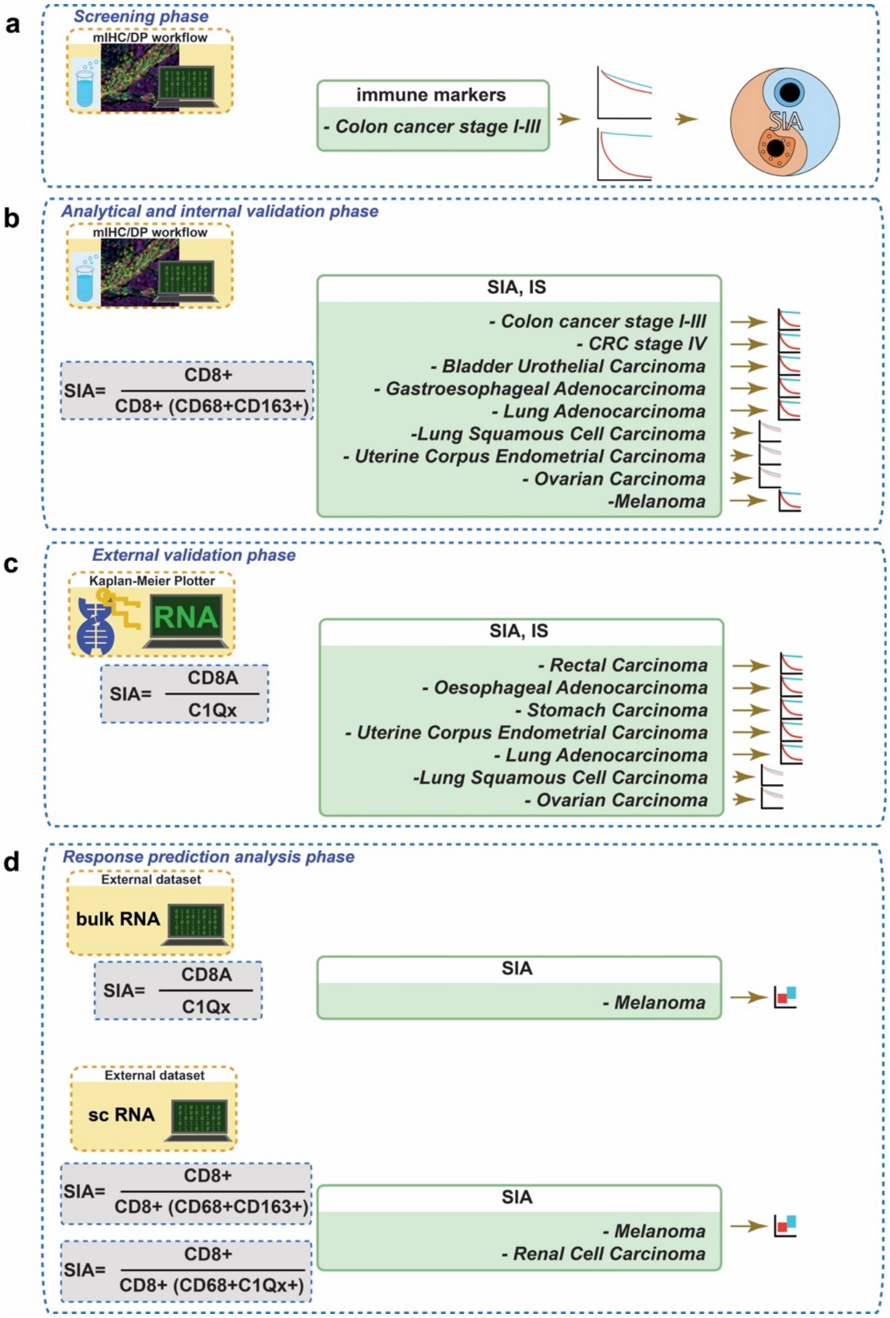

Supplementary Figure 1 Study schematics. (a) Screening phase was implemented using colon cancer stage I-III tissue collection and mIHC/DP workflow. (b) Analytical and internal validation phase was implemented by applying mIHC/DP workflow in several independent cancer tissue collections. (c) In the External validation phase the KM plotter database of the bulk RNA expression was used to estimate the impact on survival in several cancers. (d) The datasets with bulk RNA expression and single cell RNA data were used to estimate the capacity of the analysed biomarker to predict the response to the immune therapy.

Abbreviations: mIHC/DP, multiplex immunohistochemistry / digital pathology;

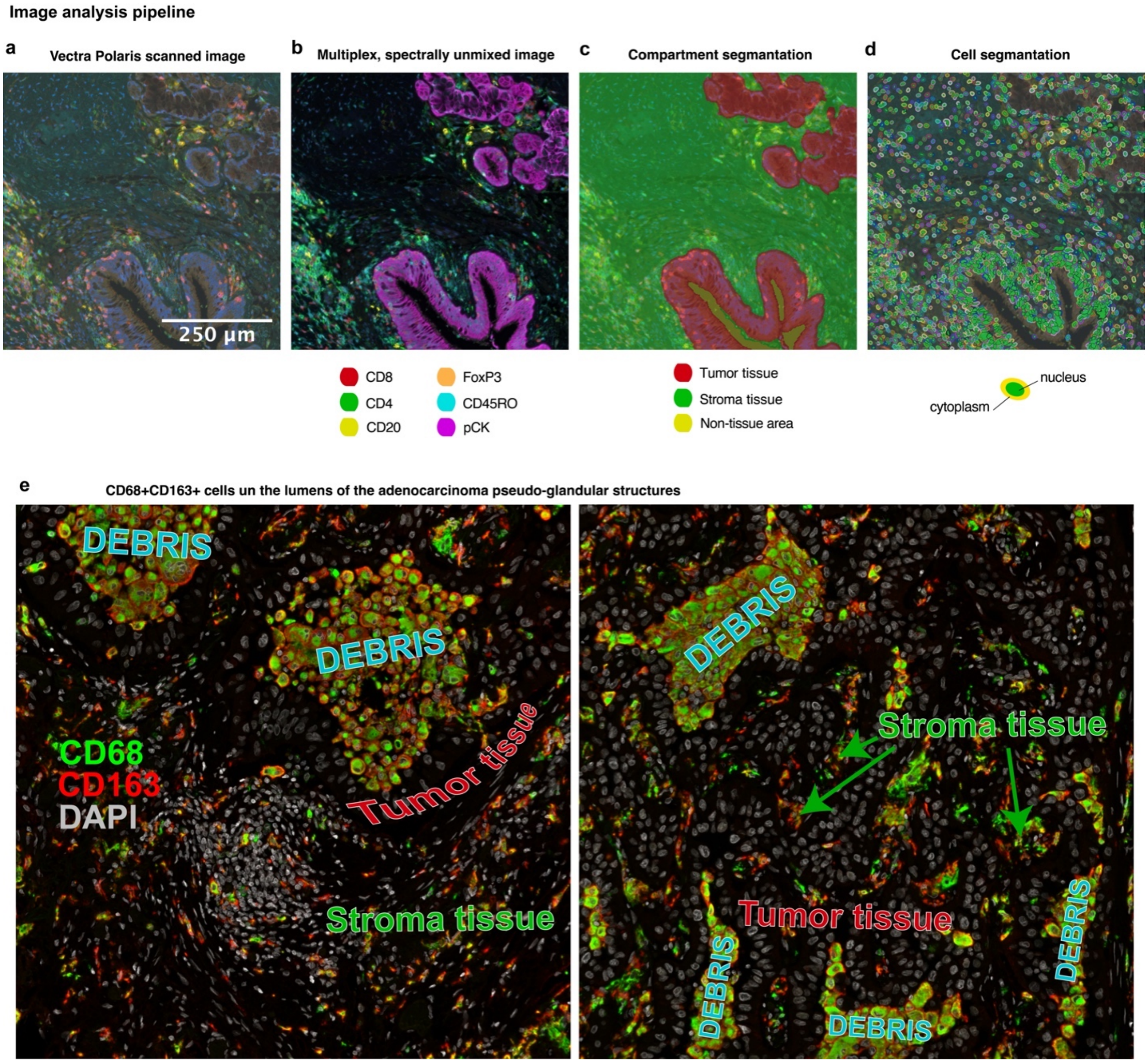

Supplementary Figure 2, Related to Figure 1. Image analysis pipeline and illustration of spectral unmixing, compartment segmentation and cell segmentation. The multi-layer multispectral image (a) is processed through the spectral unmixing algorithm to generate an oligo-layer image, where each grey-scale layer corresponds to either specific marker (immune or pan-cytokeratin), or DAPI (DNA stain) or tissue autofluorescence. (b) For visualization purposes the grey-scale layers are assigned different colours, and the combined layers demonstrated as a multi-colour image. (c) The tumour tissue, stroma and non-tissue compartments are classified using a machine-learning image analysis approach, and classification results are demonstrated by colour masks. (d) The cell nuclei are segmented using DAPI staining and algorithm that considered the size, intensity and other features of DAPI-stained regions. Perinuclear regions are considered as cell cytoplasm. Nuclei are visualized as green masks and cell cytoplasm regions as perinuclear coloured masks (colours are arbitrary and only for visualization purposes). Each image is curated by a pathologist and areas of necrosis, artefacts and debris (e) are excluded.

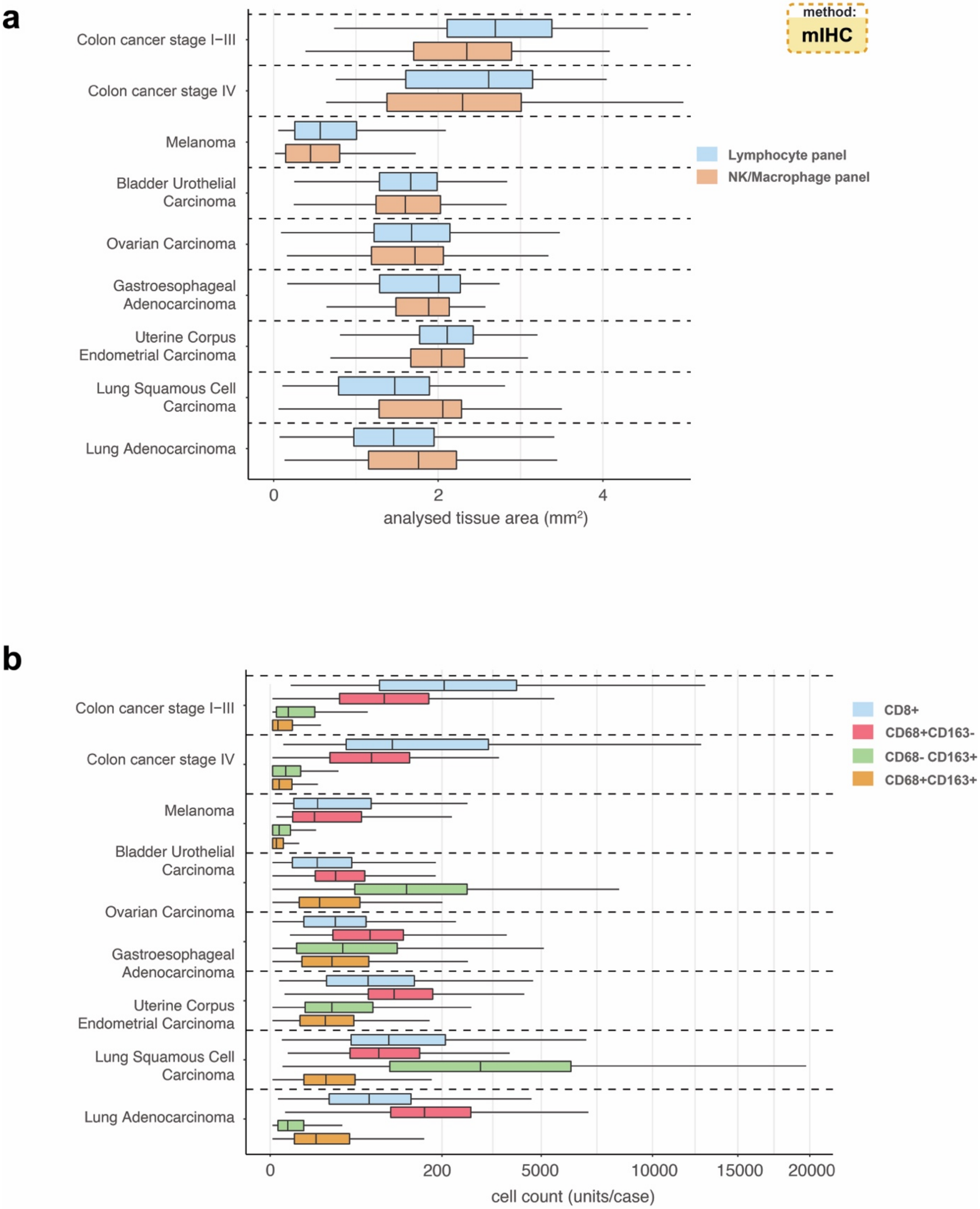

Supplementary Figure 3, Related to Figure 1. Characteristics of the analysed tissue samples from different TMA cohorts. Box-plots, representing the (a) analysed tissue are available for the analysis per patient, and (b) absolute cell counts available for the analysis per patient (horizontal axis is root-transformed). Boxes, median and interquartile range (IQR) of the ratios; whiskers, 1.5 IQR

a

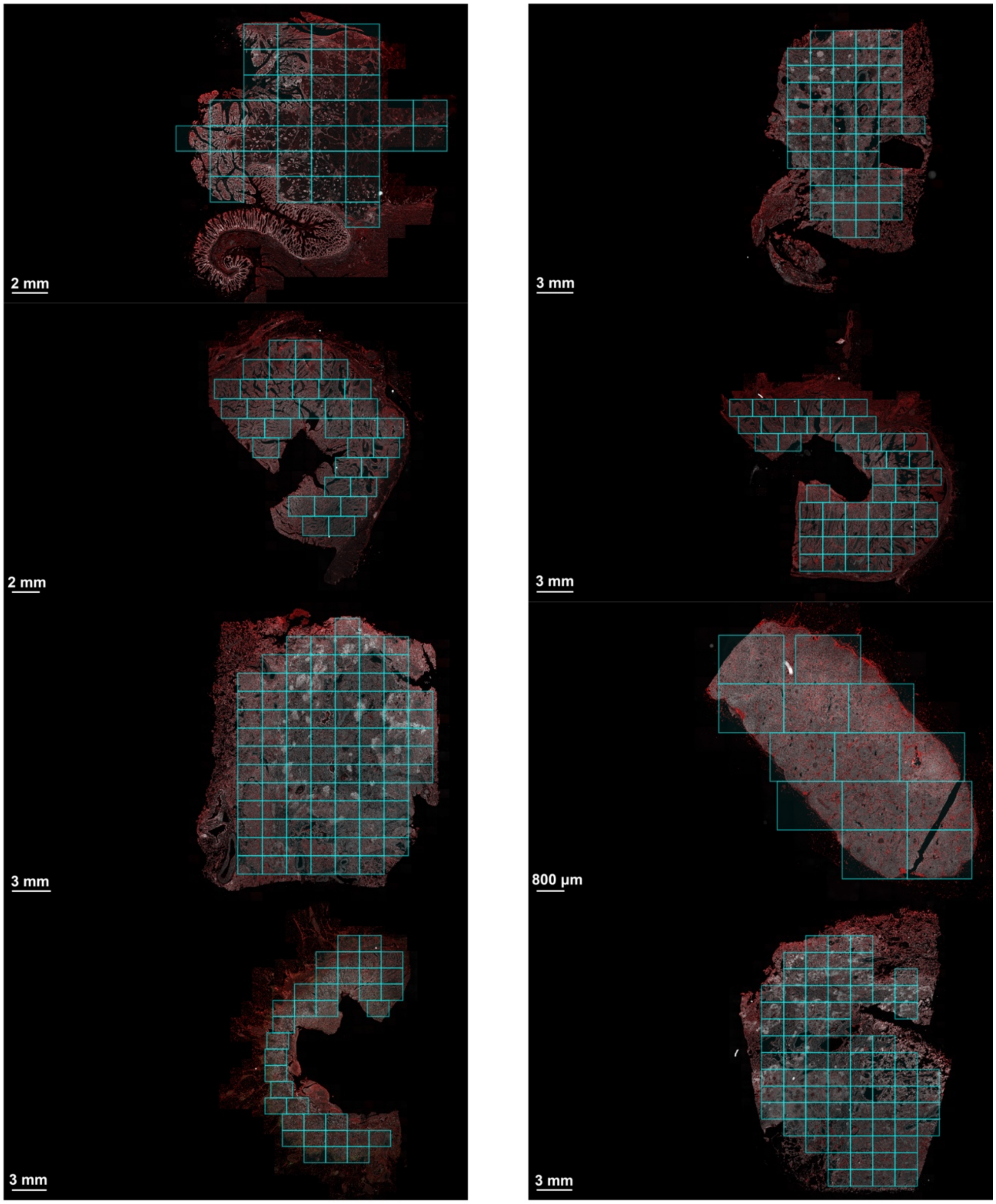
2 mm
2 mm
3 mm
3 mm
3 mm
3 mm
800 µm
3 mm

b

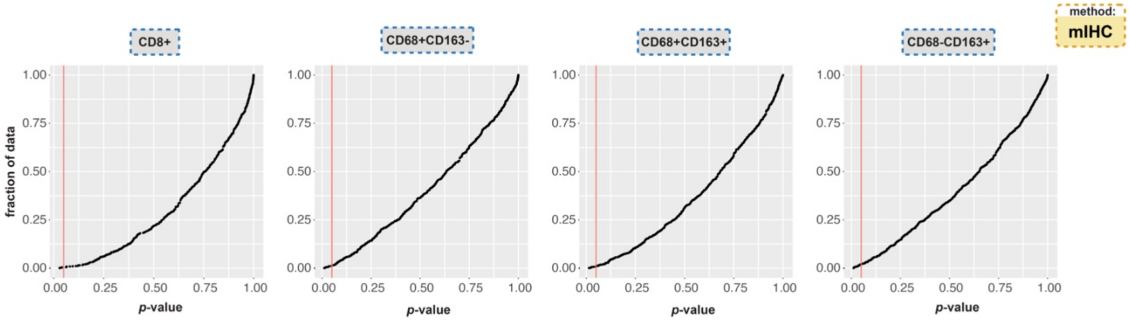
CD8+
CD68+CD163-
CD68+CD163+
CD68-CD163+
method:
mIHC
fraction of data
p-value
0.00
0.25
0.50
0.75
1.00

Supplementary Figure 4, Related to Figure 1. Analysis of the representativity of the whole slide sections by the TMA samples. (a) Digital scans of the WSS from eight tumour blocks (four from colorectal cancer and four from lung cancer) stained with antibodies against CD8, CD68 and CD163 (not visualized). Each digital WSS was applied for multispectral scanning using the ties equivalent to those applied for imaging TMA cores, illustrated as cyan rectangles. (b) The *p*-values, generated by two-sample Kolmogorov-Smirnov test repeated by 1000-times resampling are plotted as empirical cumulative distributions for each of the marker. The cut-off *p*-value=0.050 is indicated by vertical red line.

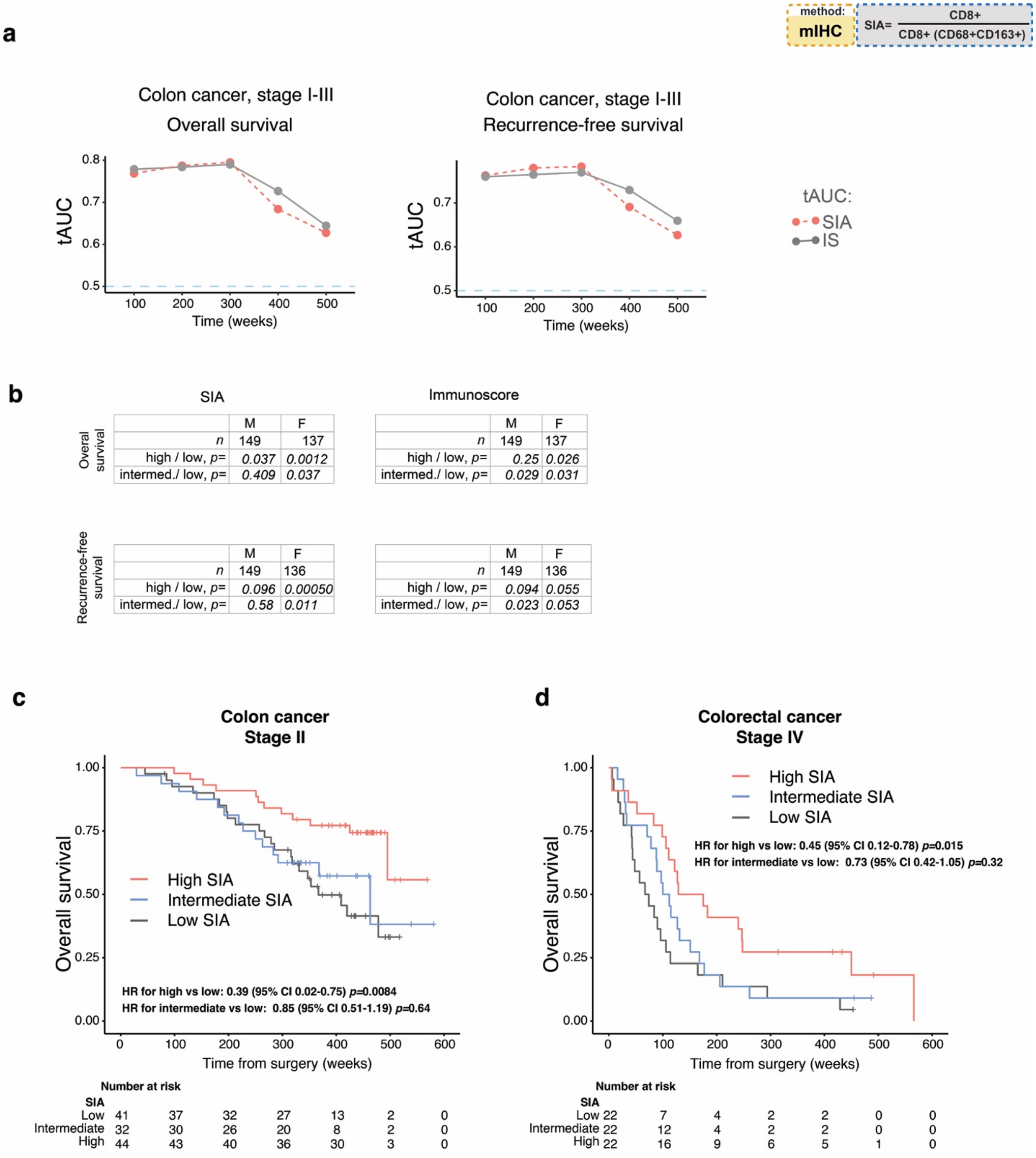

Supplementary Figure 5, Related to Figure 2. (a) Predictive accuracy of SIA and IS for OS and PFS in colon cancer stage I-III, demonstrated by tAUC analysis. No statistically significant difference is found between SIA and IS at different timepoints. **(b) Predictive accuracy of SIA and IS for OS and PFS in colon cancer stage I-III, analysed in females and males. The SIA is prognostic in (c) therapy-naïve colon cancer stage II patients (n=117) and (d) metastatic colorectal cancer patients (n=66)**. Kaplan-Meier curves and numbers at risk demonstrate OS in patient groups, stratified by trichotomized SIA. Cox proportional hazards models were used to estimate relative hazards. SIA, signature of immune activation.

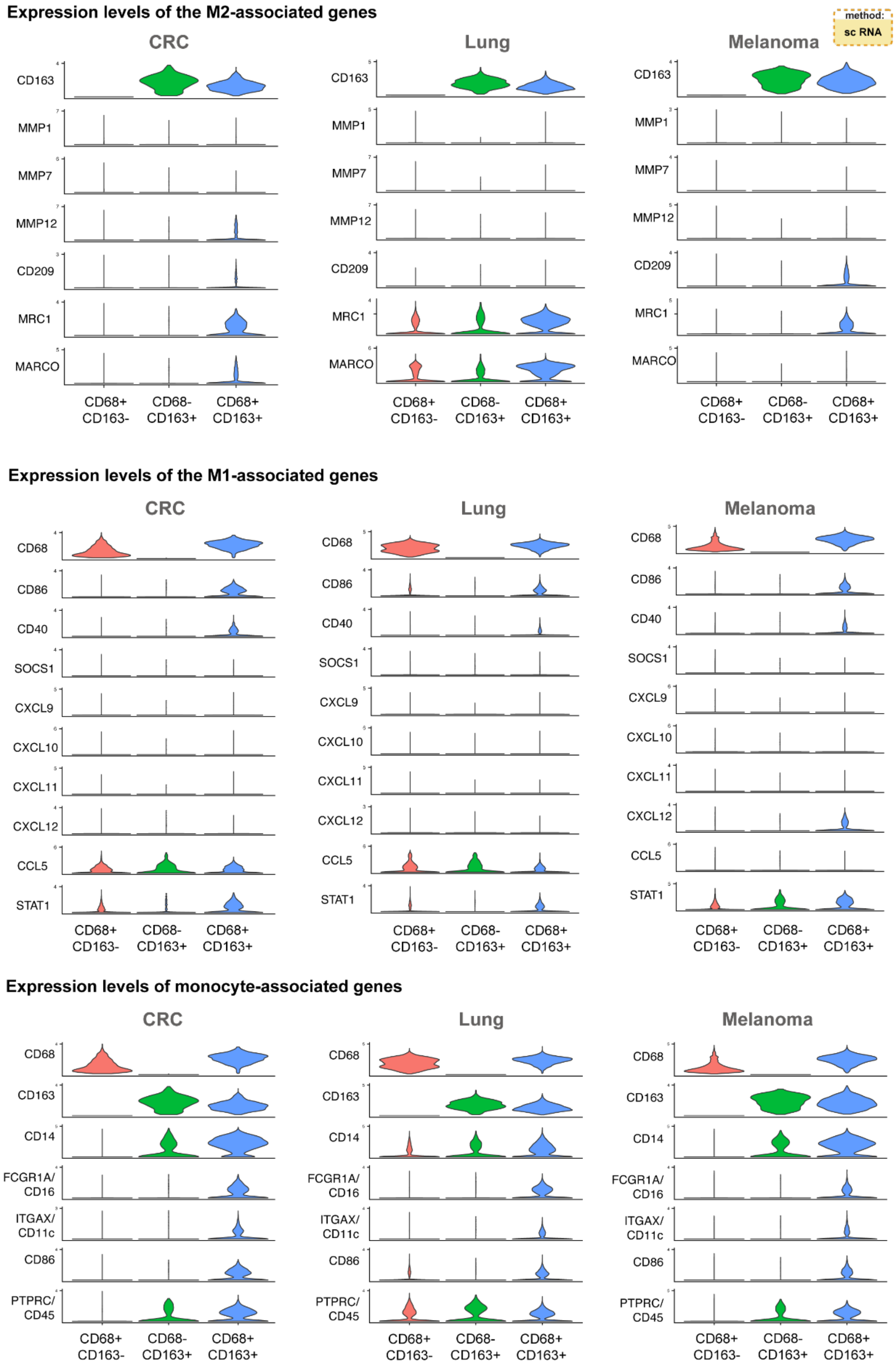

Supplementary Figure 6, Related to Figure 4. Expression level distributions of macrophage type M1- and M2-associated genes.

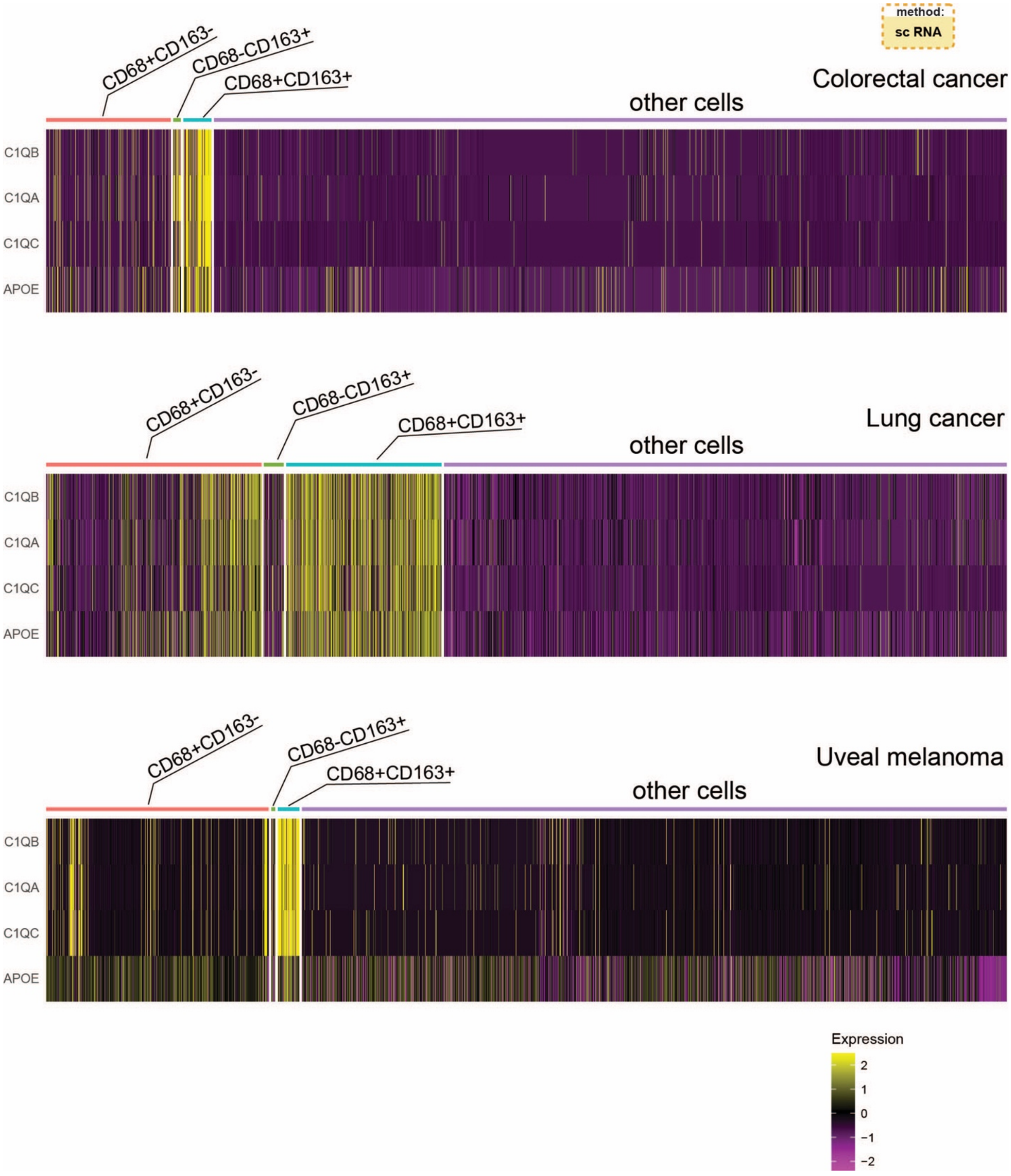

Supplementary Figure 7, Related to Figure 4. Complement complex C1q expression is a hallmark of M2-like macrophages in the three tumour types. Heatmap of scaled gene expression values of C1QA, C1QB, C1QC and APOE within M1-like, M2-like and $CD68^{-}CD163^{+}$ macrophage classes and in other cells in the single-cell RNA sequencing datasets from colon cancers (42), lung cancers (43) and uveal melanoma (44).

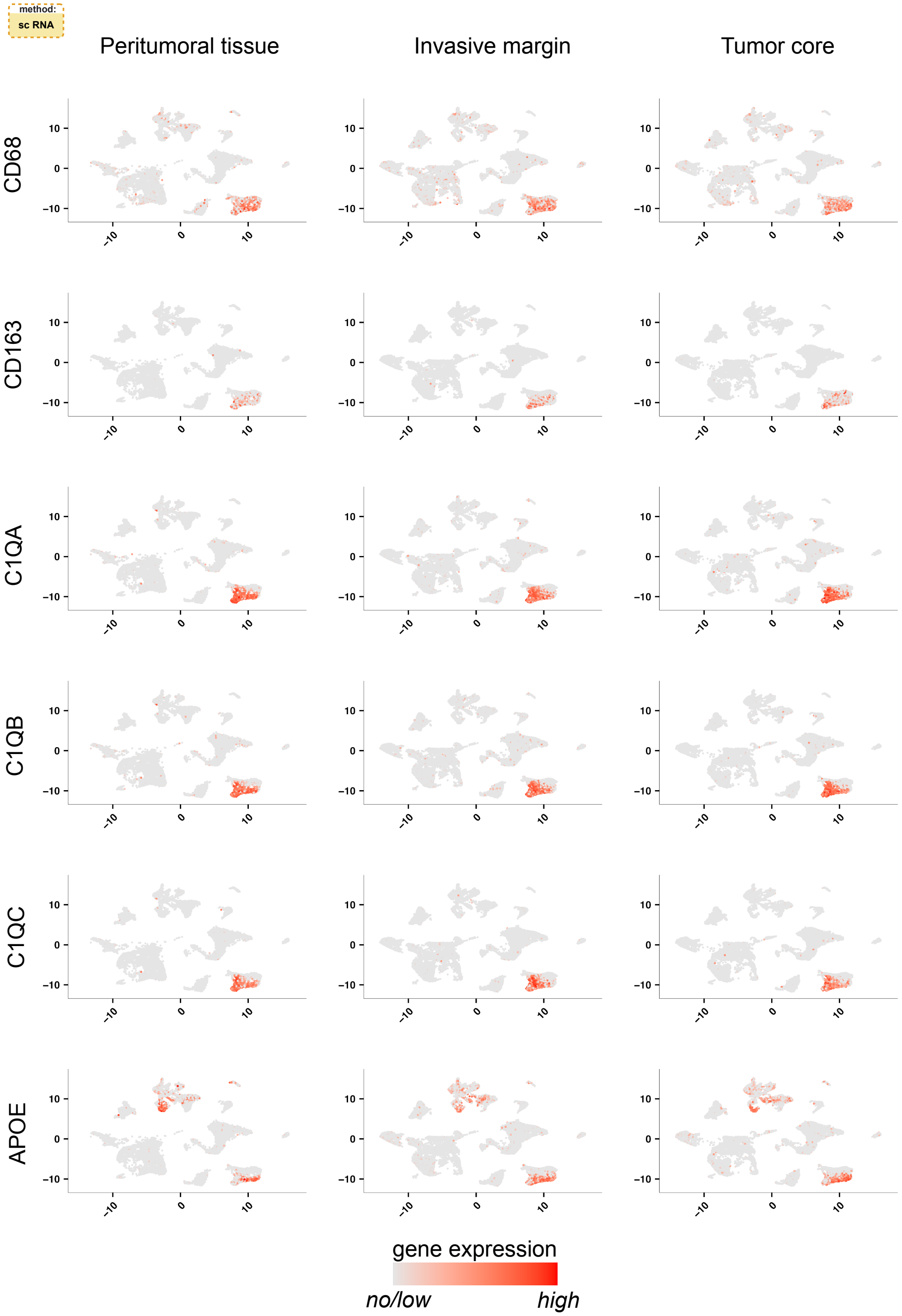

Supplementary Figure 8, Related to Figure 4. Complement complex C1q and APOE expression level is similar in different regions of colorectal cancer and peritumoral tissue. tSNE plots demonstrating the clustering of the cells from the colorectal cancer single-cell RNA sequencing dataset (42) split into three groups according to the location of the collected sample. The cells coloured according to the scaled expression values are analysed genes (CD68, CD163, C1QA, C1QB, C1QC and APOE).

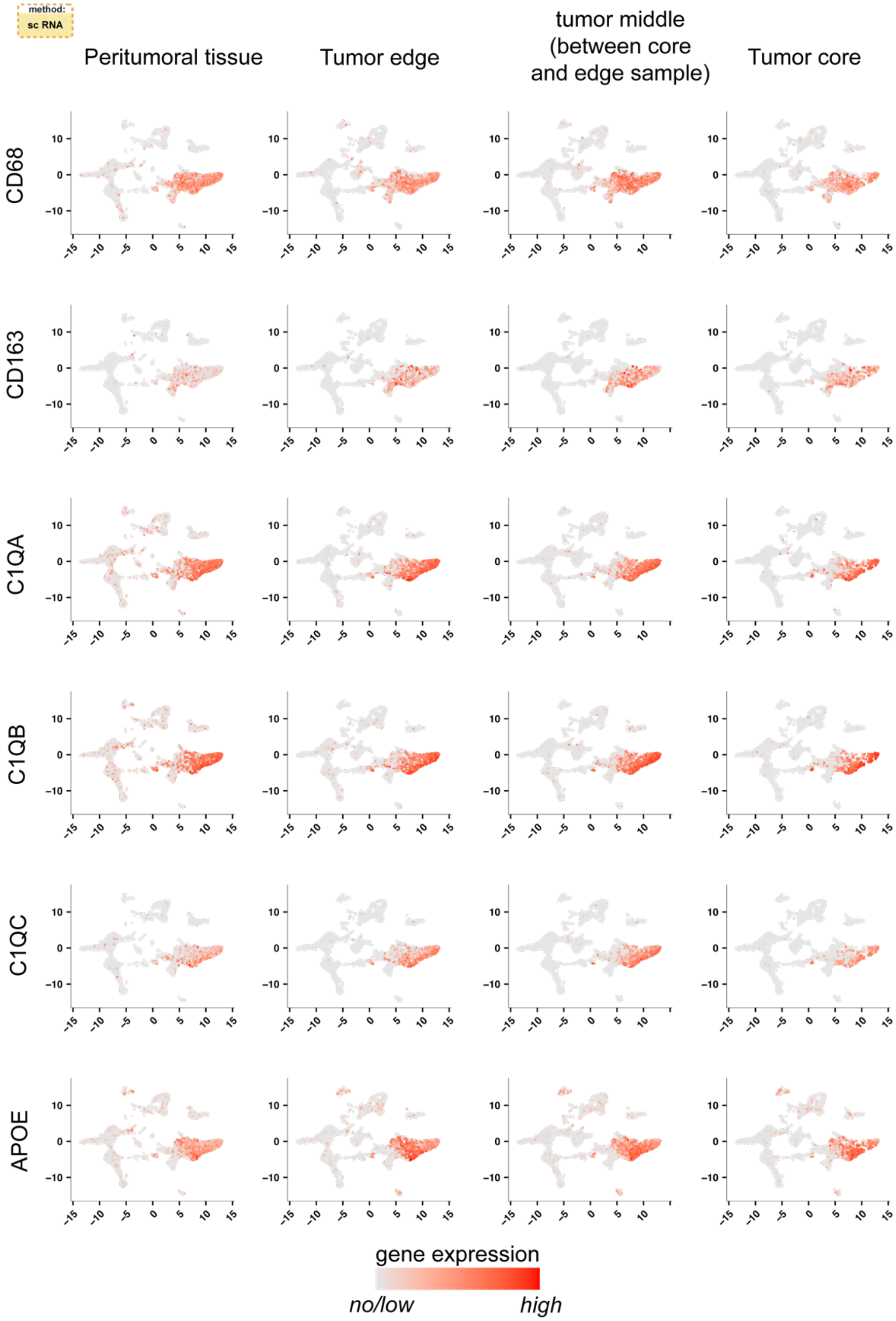

Supplementary Figure 9, Related to Figure 4. Complement complex C1q and APOE expression level is similar in different regions of lung cancer and peritumoral tissue. tSNE plots demonstrating the clustering of the cells from the lung cancer single-cell RNA sequencing dataset (43) split into three groups according to the location of the collected sample. The cells coloured according to the scaled expression values are analysed genes (CD68, CD163, C1QA, C1QB, C1QC and APOE).

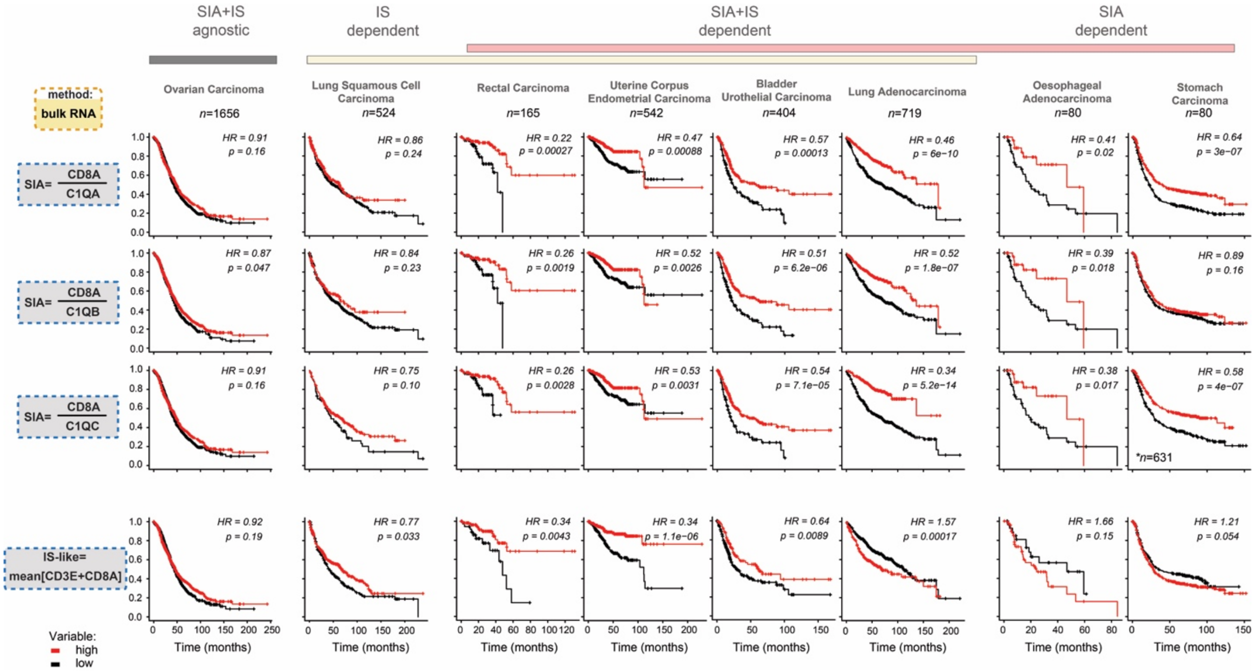

Supplementary Figure 10, Related to Figure 4. The bulk-RNA-derived SIA is prognostic in bladder cancer, oesophageal adenocarcinoma, stomach adenocarcinoma, lung adenocarcinoma and melanoma. Overall survival stratified by dichotomized ratio between the bulk RNA expression levels of CD8A and each of C1q complement subunits: C1QA, C1QB and C1QC in seven tumour types (three upper panels) and overall survival stratified by dichotomized average bulk RNA expression levels of CD8A and CD3E (IS-like metric). Gene expression and survival data was achieved from the KM plotter database: bladder, oesophageal, rectal, endometrial cancers (48), ovarian cancer (49), gastric cancer (50), lung adenocarcinoma and lung squamous cell carcinoma (51).

Supplementary Tables

Supplementary Table 1, Related to Figure 1. Baseline clinicopathological characteristics of the colon cancer cohort used for univariate associations of tissue immune cell densities and survival. Patient data shown for cases where successful staining was available from the Lymphocyte panel, the NK/Macrophage panels. Values are shown as the number of cases (percentages) unless indicated otherwise. Percentages may not add to 100% due to rounding. MSI, microsatellite instability; MMR, mismatch repair;

**Characteristics**	Lymphocyte panel (*n* = 298)	NK/Macrophage panel (*n* = 288)
**Age**		
Average age in years ± SD	69.97 ± 12.70	69.98 ± 12.75
≤ 75 years old	187 (62.8%)	179 (62.2%)
> 75 years old	111 (37.2%)	109 (37.8%)
**Sex**		
Male	154 (51.7)	139 (48.3)
Female	144 (48.3)	149 (51.7)
**pT Stage**		
0	0	0
1	26 (8.7%)	24 (8.3%)
2	16 (5.4%)	15 (5.2%)
3	203 (68.1%)	199 (69.1%)
4	53 (17.8%)	50 (17.4%)
Missing data	0	0
**pN Stage**		
0	157 (52.7%)	151 (52.4%)
1+	140 (47%)	136 (47.2%)
Missing data	1 (0.3%)	1 (0.3%)
**pM Stage**		
0	296 (99.3%)	286 (99.3%)
1	0	0
Missing data	2 (0.7%)	2 (0.7%)
**pTNM stage**		
0	0	0
1	37 (12.4%)	34 (11.8%)
2	121 (40.6%)	118 (41%)
3	140 (47%)	136 (47.2%)
4	0	0
Missing data	0	0

**Differentiation Grade**		
Low	220 (73.8%)	212 (73.6%)
High	47 (15.8%)	46 (16%)
Missing data	31 (10.4%)	30 (10.4%)
**Neural Invasion**		
No	228 (76.5%)	219 (76%)
Yes	36 (12.1%)	35 (12.2%)
Missing data	34 (11.4%)	34 (11.8%)
**Vascular Invasion**		
No	202 (67.8%)	195 (67.7%)
Yes	71 (23.8%)	68 (23.6%)
Missing data	25 (8.4%)	25 (8.7%)
**MSI/MMR Status**		
MMR proficient	237 (79.5%)	228 (79.2%)
MMR deficient	53 (17.8%)	51 (17.7%)
Missing data	8 (2.7%)	9 (3.1%)
**BRAF Mutation**		
No	139 (46.6%)	133 (46.2%)
Yes	34 (11.4%)	33 (11.5%)
Missing data	125 (41.9%)	122 (42.4%)

Supplementary Table 2, Related to Figure 2. Baseline clinicopathological characteristics of the colorectal cancer sub-cohorts used for SIA analysis. Patient data shown for cases where successful staining was available from both Lymphocyte panel and NK/Macrophage panels and SIA was calculated. Values are shown as the number of cases (percentages) unless indicated otherwise. Percentages may not add to 100% due to rounding. MSI, microsatellite instability; MMR, mismatch repair;

**Characteristics**	Colon cancer stage I-III, therapy-naïve (*n* = 286)	Colon cancer stage II, therapy-naïve (*n* = 117)	Colorectal cancer stage IV, therapy-naïve (*n* = 66)
**Age**			
Average age in years ± SD	70.01 ± 12.73	70.98 ± 13.46	70.38 ± 11.01
≤ 75 years old	178 (62.2%)	70 (59.8%)	41 (62.1%)
> 75 years old	108 (37.8%)	47 (40.2%)	25 (37.9%)
**Sex**			
Male	149 (52.1%)	57 (48.7%)	36 (54.5%)
Female	137 (47.9%)	60 (51.3%)	30 (45.5%)
**Location**			
Colon	286 (100%)	117 (100%)	61 (92.4%)
Rectum	0	0	5 (7.6%)
**pT Stage**			
0	0	0	0
1	24 (8.4%)	0	2 (3%)
2	15 (5.2%)	0	1 (1.5%)
3	198 (69.2%)	98 (83.8%)	26 (39.4%)
4	49 (17.1%)	19 (16.2%)	33 (50%)
Missing data	0	0	4 (6.1%)
**pN Stage**			
0	150 (52.4%)	117 (100%)	14 (21.2%)
1+	135 (47.2%)	0	52 (78.8%)
Missing data	1 (0.3%)	0	0
**pM Stage**			
0	284 (99.3%)	116 (99.1%)	0
1	0	1 (0.9%)	66 (100%)
Missing data	2 (0.7%)	0	0
**pTNM stage**			
0	0	0	0
1	34 (11.9%)	0	0
2	117 (40.9%)	117 (100%)	0
3	135 (47.2%)	0	0

4	0	0	66 (100%)
Missing data	0	0	0
**Differentiation Grade**			
Low	211 (73.8%)	101 (86.3%)	29 (43.9%)
High	45 (15.7%)	13 (11.1%)	23 (34.8%)
Missing data	30 (10.5%)	3 (2.6%)	14 (21.2%)
**Neural Invasion**			
No	217 (75.9%)	98 (83.8%)	29 (43.9%)
Yes	35 (12.2%)	7 (6%)	24 (36.4%)
Missing data	34 (11.9%)	12 (10.3%)	13 (19.7%)
**Vascular Invasion**			
No	193 (67.5%)	92 (78.6%)	21 (31.8%)
Yes	68 (23.8%)	15 (12.8%)	32 (48.5%)
Missing data	25 (8.7%)	10 (8.5%)	13 (19.7%)
**MSI/MMR Status**			
MMR proficient	228 (79.7%)	86 (73.5%)	56 (84.8%)
MMR deficient	50 (17.5%)	28 (23.9%)	8 (12.1%)
Missing data	8 (2.8%)	3 (2.6%)	2 (3%)
**BRAF Mutation**			
No	132 (46.2%)	49 (41.9%)	40 (60.6%)
Yes	33 (11.5%)	10 (8.5%)	11 (16.7%)
Missing data	121 (42.3%)	58 (49.5%)	15 (22.7%)

Supplementary Table 3, Related to Figure 3. Baseline clinicopathological characteristics of validation cohorts. Data from cases where SIA could be computed. Values are shown as the number of cases (percentages) unless indicated otherwise. Percentages rounded to one decimal. *Median survival times were calculated using the Kaplan-Meier method. Mean survival times were estimated when median survival times cannot be calculated from the data.

	Endometrial Cancer	Lung Adenocarcinoma	Lung Squamous Cell Carcinoma	Urine Bladder Cancer	Ovarian Cancer	Gastro-oesophageal Cancer	Melanoma
**Patient sample size and median survival time**							
Total number of patients (%)	295 (26.1%)	163 (13.1%)	89 (7.2%)	224 (19.8%)	141 (12.5%)	127 (11.2%)	94 (8.3%)
Median survival time* ± SD (weeks)	545.08 ± 14.19[b]	252 ± 43.44	324 ± 67.01	348 ± 50.85	151 ± 18.55	113 ± 20.80	478.4 ± 56.96
**Age at diagnosis**							
Mean ± SD	66 ± 10.47	66.71 ± 7.40	68.31 ± 7.50	70.69 ± 11.81	63.70 ± 8.39	70.8 ± 11.04	60.45 ± 14.61
Median ± SD	66 ± 10.47	66 ± 7.40	68 ± 7.50	72 ± 11.81	63 ± 8.39	72 ± 11.04	61.09 ± 14.61
≤ Median	152 (51.5%)	83 (50.9%)	45 (50.6%)	115 (51.3%)	73 (51.8%)	64 (50.4%)	47 (50%)
> Median	143 (48.5%)	80 (49.1%)	44 (49.4%)	109 (48.7%)	68 (48.2%)	63 (49.6%)	47 (50%)
**Sex**							
Female	295 (100%)	90 (55.2%)	38 (42.7%)	50 (22.3%)	141 (100%)	29 (22.8%)	44 (46.8%)
Male	-	73 (44.8%)	51 (57.3%)	174 (77.7%)	-	98 (77.2%)	50 (53.2%)
**pT classification**							
pT0	-	-	-	102 (45.5%)	-	0	0
pT1	-	-	-	95 (42.4%)	-	7 (5.5%)	23 (24.5%)
pT2	-	-	-	18 (8%)	-	22 (17.3%)	27 (28.7%)
pT3	-	-	-	7 (3.1%)	-	77 (60.6%)	23 (24.5%)
pT4	-	-	-	2 (0.9%)	-	21 (16.5%)	15 (16%)
Missing data	295 (100%)	163 (100%)	89 (100%)	0	141 (100%)	0	6 (6.4%)
**pN classification**							
pN0	-	-	-	24 (10.7%)	-	36 (28.3%)	-
pN1	-	-	-	5 (2.2%)	-	26 (20.5%)	-
pN2	-	-	-	0	-	33 (26%)	-
pN3	-	-	-	0	-	32 (25.2%)	-
Missing data	295 (100%)	163 (100%)	89 (100%)	195 (87.1%)	141 (100%)	0	94 (100%)
**pM classification**							
pM0	-	-	-	60 (26.8%)	-	116 (91.3%)	-
pM1	-	-	-	18 (8%)	-	3 (2.4%)	-

pM2	-	-	-	0	-	8 (6.3%)	-
Missing data	295 (100%)	163 (100%)	89 (100%)	146 (65.2%)	141 (100%)	0	94 (100%)
**Clinical stage at diagnosis**							
1	242 (82%)	155 (61.8%)	100 (61.3%)	57 (64%)	23 (16.3%)	-	-
2	8 (2.7%)	53 (21.1%)	27 (16.6%)	26 (29.2%)	16 (11.3%)	-	-
3	39 (13.2%)	36 (14.3%)	30 (18.4%)	6 (6.7%)	72 (51.1%)	-	-
4	6 (2%)	7 (2.8%)	6 (3.7%)	0	19 (13.5%)	-	-
Missing data	0	0	0	0	11 (7.8%)	127 (100%)	94 (100%)
**Differentiation grade / Histological differentiation**							
G1 Well differentiated (Low grade)	247 (83.7%)	-	-	71 (31.7%)	4 (2.8%)	77 (60.6%)	-
G2 Moderately differentiated (Intermediate grade)	-	-	-	-	31 (22%)	-	-
G3 Poorly differentiated (High grade)	48 (16.3%)	-	-	153 (68.3%)	106 (75.2%)	50 (39.4%)	-
Missing data	0	163 (100%)	89 (100%)	0	0	0	94 (100%)
**WHO performance status**							
0	-	104 (63.8%)	50 (56.2%)	-	-	-	-
1	-	56 (34.4%)	39 (43.8%)	-	-	-	-
2	-	3 (1.8%)	0	-	-	-	-
≥ 3	-	0	0	-	-	-	-
Missing data	295 (100%)	0	0	224 (100%)	141 (100%)	127 (100%)	94 (100%)
**Resection margin**							
R0	-	-	-	-	-	85 (66.9%)	-
R1	-	-	-	-	-	35 (27.6%)	-
R2	-	-	-	-	-	7 (5.5%)	-
Missing data	295 (100%)	163 (100%)	89 (100%)	224 (100%)	141 (100%)	0	94 (100%)
**p53 status**							
Mutant	32 (10.8%)	-	-	-	-	-	-
Wild-type	263 (89.2%)	-	-	-	-	-	-
Missing data	0	163 (100%)	89 (100%)	224 (100%)	141 (100%)	127 (100%)	94 (100%)
**MSI/MSS status**							
MSI	-	-	-	-	2 (1.4%)	11 (8.7%)	-
MSS	-	-	-	-	136 (96.5%)	116 (91.3%)	-
Missing data	295 (100%)	163 (100%)	89 (100%)	224 (100%)	3 (2.1%)	0	94 (100%)
**Neural invasion**							
Yes	-	-	-	-	-	9 (7.1%)	-

No	-	-	-	-	-	22 (17.3%)	-
Missing data	295 (100%)	163 (100%)	89 (100%)	224 (100%)	141 (100%)	96 (75.6%)	94 (100%)
**Vascular invasion**							
Yes	-	-	-	-	-	10 (7.9%)	-
No	-	-	-	-	-	41 (32.3%)	-
Missing data	295 (100%)	163 (100%)	89 (100%)	224 (100%)	141 (100%)	76 (59.8%)	94 (100%)
**Smoking history**							
Current smoker	-	144 (88.3%)	84 (94.4%)	92 (41.1%)	-	-	-
Non-current smoker	-	19 (11.7%)	5 (5.6%)	18 (8%)	-	-	-
Missing data	295 (100%)	0	0	114 (50.9%)	141 (100%)	127 (100%)	94 (100%)
**Adjuvant treatment**							
Yes	270 (91.5%)	-	-	69 (30.8%)	70 (49.6%)	10 (7.9%)	-
No	25 (8.5%)	-	-	0	1 (0.7%)	117 (92.1%)	-
Missing data	0	163 (100%)	89 (100%)	115 (69.2%)	70 (49.6%)	0	94 (100%)
**Neoadjuvant treatment**							
Yes	-	0	0	2 (0.9%)	-	-	-
No	-	251 (100%)	89 (100%)	0	-	-	-
Missing data	295 (100%)	0		222 (99.1%)	141 (100%)	127 (100%)	94 (100%)

Supplementary Table 4, Antibodies and amplification reagents used for multiplex fluorescence IHC.

Staining protocols were established for the lymphocyte and the NK/macrophage panel, respectively. The staining procedure included 5 to 6 cycles of microwave treatment, incubation with primary antibody, and amplification system fluorophore labelling. The final cycle was followed by DAPI staining and slide mounting. *Antigen retrieval performed in microwave oven at 100 °C, 15min. †Amplification systems ImmPRESS® HRP or Opal HRP were used: The ImmPRESS® HRP Anti-Mouse IgG (Peroxidase) (Cat. No: MP-7402-50) and Anti-Rabbit IgG (Peroxidase) Polymer Detection Kits, made in Horse (Cat No: MP-7401-50) (Vector Laboratories); Opal™ Polymer anti-Rabbit+anti-Mouse HRP Kit (Cat No: ARH1001EA) (Akoya). #in melanoma instead of cytokeratin/E-cadherin cocktail, Melan A was used to identify malignant tissue vs stroma.

Panel	Order	Antigen retrieval*	Marker	RRID	Clone	Host Species	Dilution	Amplification/enzyme reagent†	Company
Lymphocyte panel	1.	pH9	CD8a	AB_11150240	C8/144B	Mouse	1:250	ImPress	Thermo Fisher
	2.	pH9	CD4	AB_2728838	4B12	Mouse	1:100	ImPress	Agilent
	3.	pH6	CD20	AB_2282030	L26	Mouse	1:1500	Opal HRP	Agilent
	4.	pH6	FoxP3	AB_2797979	D6O8R	Rabbit	1:300	ImPress	Cell Signaling
	5.	pH6	CD45RO	AB_1072483	UCHL1	Mouse	1:400	Opal HRP	Thermo Fisher
	6.	pH6	PanCK	AB_306047	C-11	Mouse	1:100	Opal HRP	Abcam
			Cytokeratin	AB_2132885	AE1/AE3	Mouse	1:400		Agilent
			E-cadherin	AB_397581	36/E	Mouse	1:2000		BD Biosciences
	6.#	pH6	Melan A	AB_2335691	A103	Mouse	1:100	ImPress	Agilent
	7.	-	DAPI	-	-	-	-	-	Akoya
NK/Macrophage panel	1.	pH6	CD3	AB_2631163	F7.2.38	Mouse	1:80	ImPress	Agilent
	2.	pH6	CD56	AB_2750583	123C3	Mouse	1:100	ImPress	Agilent
	3.	pH6	NKp46	AB_2746835	NCR1	Rabbit	1:150	ImPress	Thermo Fisher
	4.	pH6	CD68	AB_2074844	PG-M1	Mouse	1:100	Opal HRP	Agilent
	5.	pH6	CD163	AB_2756375	10D6	Mouse	1:400	Opal HRP	Novocastra
	6.	pH6	PanCK	AB_306047	C-11	Mouse	1:100	Opal HRP	Abcam
			Cytokeratin	AB_2132885	AE1/AE3	Mouse	1:500		Agilent
			E-cadherin	AB_397581	36/E	Mouse	1:2000		BD Biosciences
	6.#	pH6	Melan A	AB_2335691	A103	Mouse	1:100	ImPress	Agilent
	7.	-	DAPI	-	-	-	-	-	Akoya
